# Non-pharmacological traditional Chinese medicine interventions for depression: bibliometric mapping of Chinese databases and WoSCC, 2000–2025

**DOI:** 10.3389/fmed.2026.1964791

**Published:** 2026-10-08

**Authors:** Sihan Dong, Yazhen Lian, Jiahui Xie, Sihui Cao, Jingjing Yang, Fang Qi, Qiong Liu

**Affiliations:** Department of Traditional Chinese Medicine, School of Acupuncture-Moxibustion, Tuina and Rehabilitation, Hunan University of Chinese Medicine, Changsha, Hunan, China

**Keywords:** acupuncture, bibliometrics, depression, network analysis, research trends, traditional Chinese medicine

## Abstract

**Objective:**

The aim of the study was to characterize publication activity, collaboration structures, keyword themes, and temporal changes in the literature on non-pharmacological traditional Chinese medicine interventions for depression.

**Methods:**

Journal records published from 2000 to 2025 were obtained from CNKI, Wanfang, VIP, and the Web of Science Core Collection (WoSCC). Eligibility combined mechanical rules with independent paired assessment of 2,089 boundary records and reference-based reconciliation. The final corpora contained 5,385 Chinese and 721 WoSCC records. Full-counting collaboration and keyword networks were analyzed using weighted Louvain communities, cosine-distance silhouettes, two-state burst intervals, and resolution, seed, Leiden, and terminology sensitivity analyses. Selected graph quantities were cross-checked in R/igraph. Additional checks evaluated record-identity consistency, linkage sensitivity, and annual keyword availability denominators.

**Results:**

Chinese output peaked at 390 records in 2021, while WoSCC output reached 118 records in 2025. Complete author networks contained 12,141 Chinese and 2,933 WoSCC nodes and were sparse (densities 0.000410 and 0.003572). Eligible keyword networks contained 569 Chinese and 81 WoSCC terms. Weighted Louvain yielded five Chinese communities (*Q* = 0.194124; mean silhouette = 0.005173) and four WoSCC communities (*Q* = 0.134767; mean silhouette = 0.029369). Stability analyses showed only moderate agreement across seeds and alternative algorithms, indicating that the communities are exploratory summaries rather than discrete topic classes. Keyword availability denominators materially altered some WoSCC community time profiles.

**Conclusion:**

The literature expanded substantially and centered on acupuncture-related interventions, poststroke depression, evidence synthesis terminology, exercise-based techniques, sleep, and experimental themes. Sparse collaboration networks and overlapping thematic interpretations indicate opportunities for broader collaboration and more consistent terminology. Bibliometric prominence does not establish efficacy, safety, mechanism, or causality.

## 1 Introduction

Depression is a common mental disorder that can impair social, occupational, and physical functioning. The World Health Organization estimates that about 332 million people suffer from depression and that 5.7% of adults are affected; global burden analyses likewise identify depressive disorders as major contributors to disability (1, 2). These burden estimates provide clinical context but are not outcomes of the present bibliometric analysis.

Acupuncture, moxibustion, auricular interventions, acupoint application, cupping, tuina, qigong, tai chi, Baduanjin, and related approaches have been investigated in depression research. A bibliometric study can describe how this literature is distributed and connected, but record frequency and network position cannot determine whether an intervention is effective or safe. Donthu and colleagues emphasize transparent source selection, data cleaning, technique choice, and interpretation in bibliometric research, while bibliometrix illustrates an established framework for reproducible science mapping (3, 4).

Prior bibliometric studies have mapped external traditional Chinese medicine (TCM) therapies, acupuncture, and broader TCM research for depression. Kong et al. analyzed external therapies across CNKI, Wanfang, WoS, and PubMed, reporting 1,672 Chinese and 441 English records (5). Song et al. analyzed 921 Web of Science Core Collection (WoSCC) records on broader TCM research through April 2024 (6). Condition-specific studies addressed acupuncture for poststroke depression and chronic pain-related depression or anxiety (7, 8), and Xiang et al. mapped WoSCC acupuncture-depression publications from 2011 to 2020 (9). Most importantly, Sun et al. recently compared 3,021 Chinese and 2,176 English acupuncture-related publications from 2000 to 2025 (10). The present study does not claim to be the first cross-language integration. Its narrower contribution includes a broader set of non-pharmacological TCM techniques, the inclusion of VIP, deliberately separate Chinese and WoSCC analyses, and record-level provenance with explicit mapping, sensitivity, and cross-engine checks. Count differences do not represent direct replications or effect comparisons.

Clinical evidence provides context but is analytically separate from the present bibliometric outcomes. Randomized evidence syntheses have evaluated acupuncture as a stand-alone comparator and as an adjunct to antidepressants, while syntheses of mind-body exercise include tai chi and qigong; unresolved heterogeneity and transferability require cautious interpretation (11, 12). Current guideline-based care emphasizes shared decision-making and established psychological, pharmacological, and structured activity options (13). Bibliometric frequency, centrality, bursts, and communities cannot determine efficacy, safety, or treatment recommendations.

The objectives were to describe annual publication output; author, institution, and WoSCC country/region collaboration; keyword co-occurrence; exploratory keyword communities; burst intervals; and temporal prevalence of terms assigned to whole-period communities. PRISMA-S informed the reporting of the literature searches (14), and the BIBLIO minimum requirements informed the reporting of the bibliometric review (15). Neither framework was used to imply protocol registration or a clinical effect-synthesis risk-of-bias assessment.

The central question was whether an acupuncture-centered pattern of research attention persisted when the scope included other non-pharmacological TCM techniques and when terminology and community-detection choices changed. We examined within-corpus prominence and robustness without ranking the two database groups as national research systems or treating bibliometric activity as clinical benefit.

## 2 Data and methods

### 2.1 Study design and data sources

This descriptive bibliometric mapping review used journal records from CNKI, Wanfang, VIP, and WoSCC. Chinese searches were performed on 15 July 2026; collection and replacement exports continued through 17 July 2026. The publication eligibility window was from 1 January 2000 to 31 December 2025. The WoSCC export, downloaded on 17 July 2026, contained English-language articles and reviews. Its interface date restriction used Index Date; eligibility was therefore checked against each record's publication year. SinoMed was not searched, so coverage does not extend to all Chinese biomedical databases. Figure 1 summarizes the analytical workflow.

**Figure 1:**
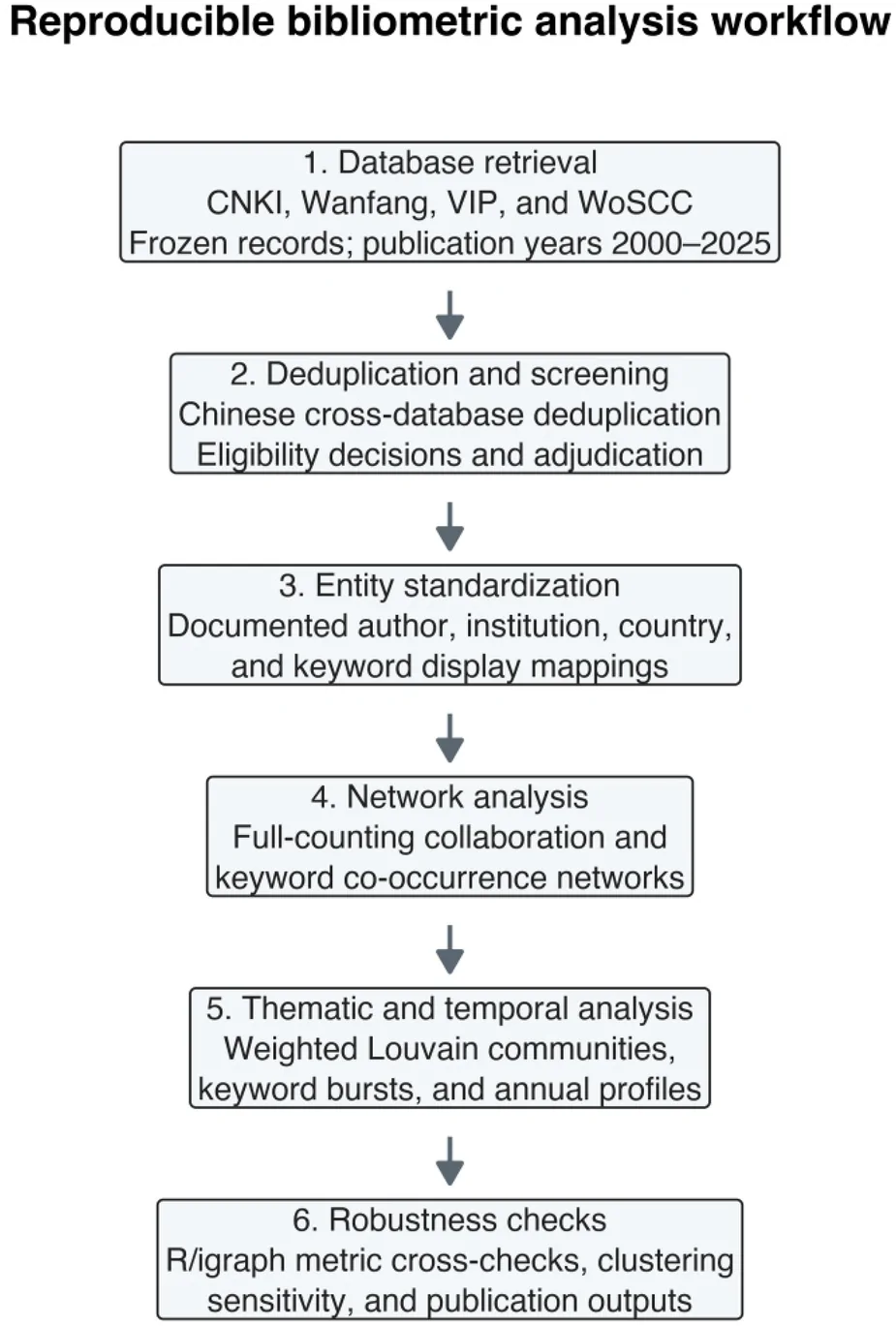
Reproducible analytical workflow from database retrieval through robustness checks.

The review was not registered, and no prospective protocol registration is claimed. The analytical unit was the bibliographic record rather than a clinical effect estimate. Accordingly, a study-level clinical risk-of-bias appraisal was not performed. Quality assurance instead targeted the review processes and data products: retained search evidence, duplicate-linkage logs, selection provenance, field completeness, normalization and mapping logs, parameter sensitivity, and independent cross-engine computational checks. The accompanying Reporting Checklist maps BIBLIO and PRISMA-S items to the current manuscript sections and Supplementary Methods, including retrieval-date and review-process limitations. The window beginning in 2000 provides a calendar-defined account of the field, and 2025 was the last complete publication year before retrieval; neither boundary denotes a demonstrated historical turning point.

### 2.2 Search strategy and reporting

The Chinese strategies combined title or keyword terms for depression with the database-specific intervention fields. Supplementary Methods S1 provides the Chinese-character expressions for CNKI, Wanfang, and VIP, the complete WoSCC topic expression, and the database editions, date fields, document types, language settings, expansion options, search dates, and export counts. Chinese search expressions used Chinese terminology; CNKI bilingual expansion supplemented these terms. No additional Chinese-language filter was applied. The WoSCC topic block was restricted to English articles or reviews. Search reporting followed PRISMA-S (14).

### 2.3 Deduplication, eligibility, and screening

Eligible records were Chinese journal articles or English WoSCC articles/reviews published during 2000–2025 in which depression and an eligible non-pharmacological TCM intervention were primary bibliographic topics. Eligibility protocol TX-01 specifies the intervention taxonomy and decision hierarchy, with worked examples (Supplementary Methods S2). Categories covered needling, thermal stimulation, non-invasive acupoint techniques, manual and surface techniques, traditional movement and breathing practices, TCM-based music, and identifiable combinations. Broad phrases such as appropriate TCM technology required an explicitly named eligible technique. Pharmacological treatment alone, incidental mentions of depression or intervention, out-of-window records, ineligible document types, confirmed duplicate versions of the same article, and records without essential identifiers were excluded. Missing abstracts or keywords alone did not exclude otherwise eligible records. The scope is technique-based: acupoint application may involve medicated preparations, and adjunctive medication was permitted. The non-pharmacological designation therefore does not mean that every included regimen was drug-free or that pharmacological contributions could be excluded.

Chinese records were linked by exact normalized DOI, exact normalized title, or normalized-title similarity of at least 0.96, with the title-based routes additionally requiring publication years to be within 1 year and an overlapping author, matching first author, or matching journal. The 14,968 exports comprised 4,757 CNKI, 5,568 Wanfang, and 4,643 VIP records; 8,705 duplicate source records were removed, leaving 6,263 records. Two reviewers assessed a random sample of 200 logged merge pairs and 200 retained-distinct candidate pairs; discordant assessments underwent reference-based reconciliation. This pair-level review examined decision consistency within the sampled candidate sets and did not estimate recall among unflagged pairs. The WoSCC export contained 1,260 unique accession numbers. Detailed matching, representative-record selection, and the original consistency review appear in Supplementary Methods S3.1–S3.2. A second-stage reference assessment of these same 400 pairs was conducted with the algorithmic decision concealed. Bibliographic identity, rather than agreement with the automatic decision, defined the reference; related publications reporting the same study were not automatically treated as the same article. The review used separate initial decisions and third-reviewer adjudication, following the distinction between false-positive and false-negative duplicates in methodological evaluations (16, 17). Final reference identities were checked across all sampled pairs: same-article links formed record-identity components, and every pair decision was derived from component membership. Stratum-specific error proportions and Wilson intervals are reported in Supplementary Methods S3.3. Separate linkage-sensitivity copies applied these constraints while keeping the included source-record set fixed (Supplementary Methods S3.4).

Records with non-empty rule-based review flags formed a boundary queue of 2,089 records (1,529 Chinese and 560 WoSCC). Flags identified ambiguous topic relevance, an intervention found only outside primary fields, incomplete metadata, and document-type or year anomalies. The other 4,734 Chinese and 700 WoSCC records followed the mechanical eligibility rules. Two reviewers independently assigned Include, Exclude, or Uncertain to the boundary records before reconciliation. Agreement was 79.37% (1,658/2,089), with Cohen's kappa = 0.623. All 437 disagreements or uncertain pairs underwent reference-based reconciliation; an additional reference check corrected four concordant decisions. The retained AI-assisted assessment had a separate agreement of 77.79% and kappa = 0.566 and was not pooled with the paired-review statistic. Final inclusion comprised 5,385 Chinese and 721 WoSCC records. Supplementary Methods S2 provides the complete decision matrix, routing counts, and reconciliation rules.

### 2.4 Entity normalization and analytical denominators

Entity normalization involved three operations: mechanical string cleaning, approved analytical mappings, and publication-only display translation. Duplicate canonical entities within a record were counted once. Author names were not merged based on initials or visual resemblance. Institution and keyword aliases changed analytical identity only when a mapping was approved and logged; other terms remained distinct. The Chinese source terms 抑郁 and 抑郁症 consequently remained separate nodes, representing a conservative non-merging decision rather than evidence of clinical non-equivalence. WoSCC Organization-Enhanced names were preferred when available. The primary WoSCC keyword analysis used Author Keywords only, without pooling Keywords Plus. English display labels did not merge or change analytical nodes. Exact operations, mapping versions, and retained-distinct boundaries are reported in Supplementary Methods S4.

Terminology sensitivity merged the broad Chinese terms 抑郁 and 抑郁症 and, separately, the WoSCC terms depression and depressive disorder, counting their union once per record. More specific conditions, including major depressive disorder and poststroke depression, retained their identities. The same frequency threshold and community-analysis settings were then applied. This evaluated the influence of broad synonym handling without asserting clinical equivalence among all depression-related terms.

A bibliometric record was the analytical unit; records included research articles and reviews and therefore must not be interpreted as independent clinical trials. Full analytical graphs retained all non-empty authors, institutions, and WoSCC countries/regions. Keyword graphs retained terms present in at least five records. Primary metrics used the complete eligible graphs. Author, institution, and country/region network displays used induced subgraphs based on the top 100 nodes plus frequency ties; keyword network and community displays used the top 50 nodes plus ties. Ranking, labels, layouts, actual displayed node counts, and the distinction between analytical and display graphs are specified in Supplementary Methods S5.

### 2.5 Network and community analysis

Full counting was used: each record contributed once to each entity and once to each unordered within-record pair. Node frequency was the number of distinct records; degree was the number of adjacent nodes; strength was the sum of incident edge weights; and density was calculated on the complete eligible graph. Normalized weighted betweenness used inverse co-occurrence weight as distance.

Weighted Louvain community detection was run at a resolution of 1.0 (18). Communities were ordered by aggregate document frequency and assigned descriptive labels from prominent terms. Separation was assessed from binary record-by-keyword profiles using cosine distances and the silhouette coefficient (19). Resolution, seed, and R/igraph Leiden sensitivity analyses evaluated dependence on analytical choices (20). Louvain assigns each keyword to a single community. Overlap refers to thematic interpretation and document coverage, rather than multiple community assignments for a keyword. Seeds, grids, and stability definitions are reported in Supplementary Methods S6.

### 2.6 Burst and temporal analysis

Keyword bursts were detected with a deterministic two-state binomial Kleinberg-style adaptation, using annual corpus totals as exposure and a minimum total frequency of five (21). The primary specification and a 3-by-3 parameter grid were evaluated. Strength is internal to this adaptation and is not comparable with CiteSpace burst strength. The emission model, dynamic program, strength definition, parameters, and sensitivity are reported in Supplementary Methods S7. Temporal profiles measured the annual prevalence of terms assigned to whole-period communities. Years with zero records have undefined prevalence and interrupt the plotted lines; open circles identify years with fewer than 10 records because proportions based on small yearly denominators are unstable. Annual keyword completeness was tabulated, and burst intervals and community time profiles were recalculated using records with available keywords as denominators (Supplementary Methods S8).

### 2.7 Cross-engine verification and citation-network scope

The custom workflow was selected to preserve source identifiers, deduplication and mapping logs, complete graph exports, explicit thresholds, deterministic layouts, and sensitivity outputs. Selected quantities were cross-checked in R/igraph using the same frozen inputs: an exact pinned Python environment passed 58 tests, and the R verifier passed 107 checks with no failures. The checks covered corpus and annual counts, nodes, edges, frequencies, degree, strength, density, components, weighted betweenness, community modularity, seed stability, and Leiden sensitivity. Existing CiteSpace workspaces used non-comparable corpora or settings and were not presented as benchmarks. Validation supports computational consistency for checked quantities but does not account for the absence of database, selection, normalization, or interpretive bias. Reproducibility identities and limitations are reported in Supplementary Methods S9.

As an additional check, all eligible keyword-node frequencies and keyword-edge weights were rebuilt from the record-level inputs and matched the primary exports exactly. The terminology sensitivity analysis tested dependence on an alternative, explicit mapping rule. The five-record keyword threshold served as a pragmatic support filter, not as an externally established optimum. A broader analysis crossed thresholds of 3, 5, and 10 with Louvain resolutions of 0.5, 1.0, and 1.5. Resolution 1.0 retains the conventional modularity scale; lower and higher settings examine aggregation and fragmentation (18, 20). An agglomerative greedy-modularity algorithm provided a further comparison at the primary threshold (22). Supplementary Methods S6 presents the parameter rationale, all 18 settings, strength-rank stability, and alternative partitions. Agreement across implementations was not interpreted as proof of unbiased selection or universally stable communities.

WoSCC cited-reference metadata were available for 720 of 721 records, whereas record-level reference lists were absent from the Chinese exports. Consequently, comparable cross-corpus cocitation or bibliographic-coupling networks could not be constructed. Adding a WoSCC-only network would address a different single-corpus question and was not undertaken (Supplementary Methods S10).

## 3 Results

### 3.1 Record flow

After deduplication and screening, 5,385 of 6,263 Chinese records and 721 of 1,260 WoSCC records were included. Exclusions totaled 878 and 539, respectively. Figure 2 reports publication-derived grouped reasons; detailed exclusion reasons remain in the retained record-level selection tables, with route totals in Supplementary Methods S2.

**Figure 2:**
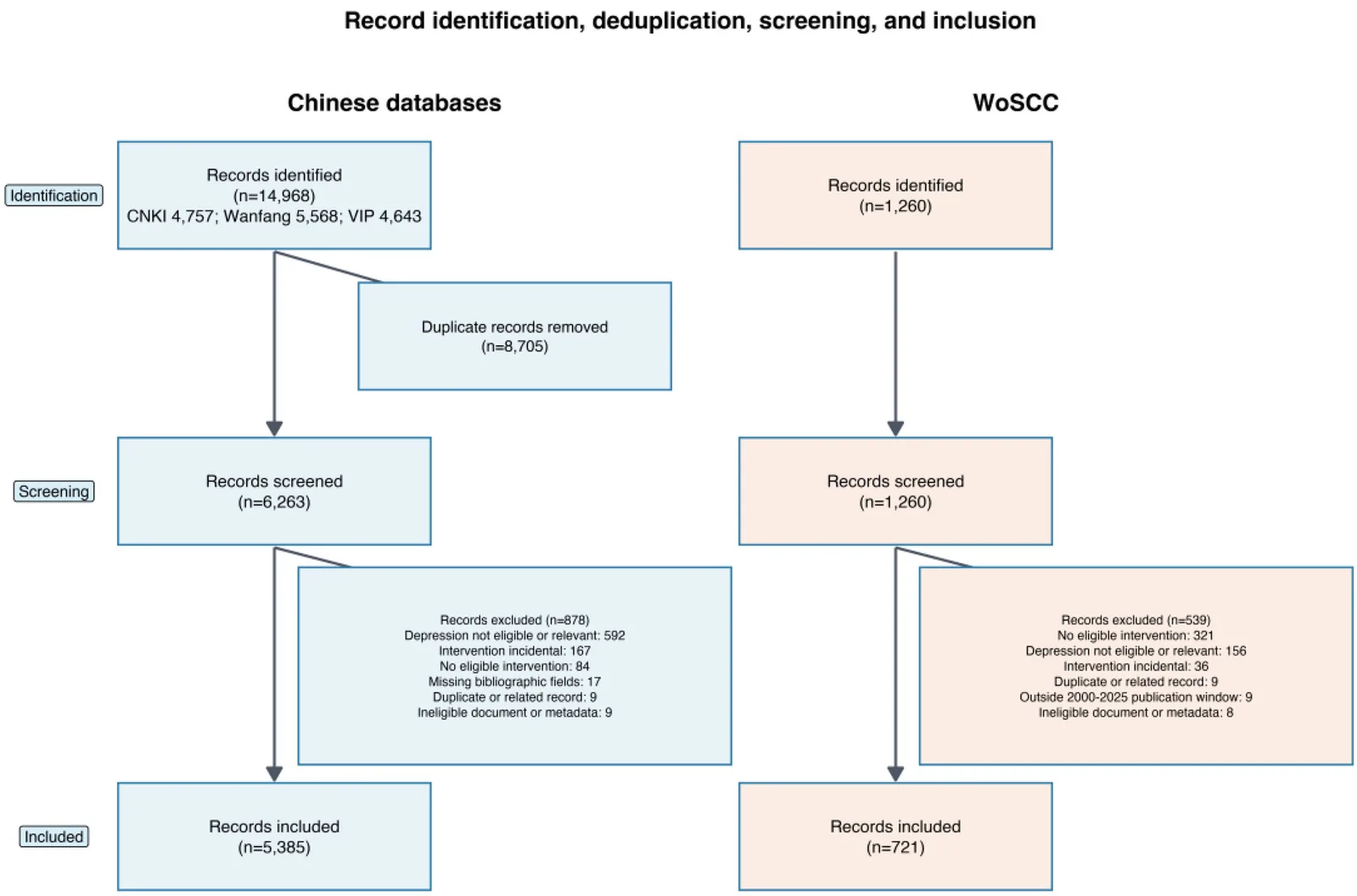
Record identification, deduplication, screening, grouped exclusion reasons, and inclusion for the Chinese and WoSCC corpora.

Within the boundary queue, 794 of 1,529 Chinese and 137 of 560 WoSCC records were included. The unflagged route contributed 4,591 of 4,734 Chinese and 584 of 700 WoSCC records. Exclusions totaled 735 + 143 = 878 and 423 + 116 = 539, respectively. The duplicate-pair review resulted in 14 reviewer disagreements among 200 merged pairs and 16 among 200 retained-distinct pairs; reference reconciliation also corrected one concordant retained-distinct assessment. These initial counts describe the consistency-review process. The separate reference assessment identified five false merges among 200 merged pairs (2.50%; 95% Wilson interval 1.07–5.72%) and 20 missed duplicates among 200 retained-distinct candidate pairs (10.00%; 6.57–14.94%). The latter represents the sampled candidate stratum, not all true duplicates, and therefore is not an overall false-negative rate. The two equally sampled strata were not pooled into a population accuracy estimate. Identity-consistency checking corrected one concordant initial classification in the retained-distinct stratum. The 5,385-record primary corpus was maintained as the descriptive baseline. Two constrained linkage-sensitivity copies contained 5,386 and 5,387 records from the same 13,393 included source records; these were not population-wide corrections (Supplementary Methods S3).

### 3.2 Annual publication output

Chinese output rose from eight records in 2000 to a maximum of 390 in 2021. WoSCC output reached 118 in 2025 (Figure 3). These curves depict indexed publication volume within the retained corpora; they do not measure clinical adoption, intervention effectiveness, or field-normalized growth.

**Figure 3:**
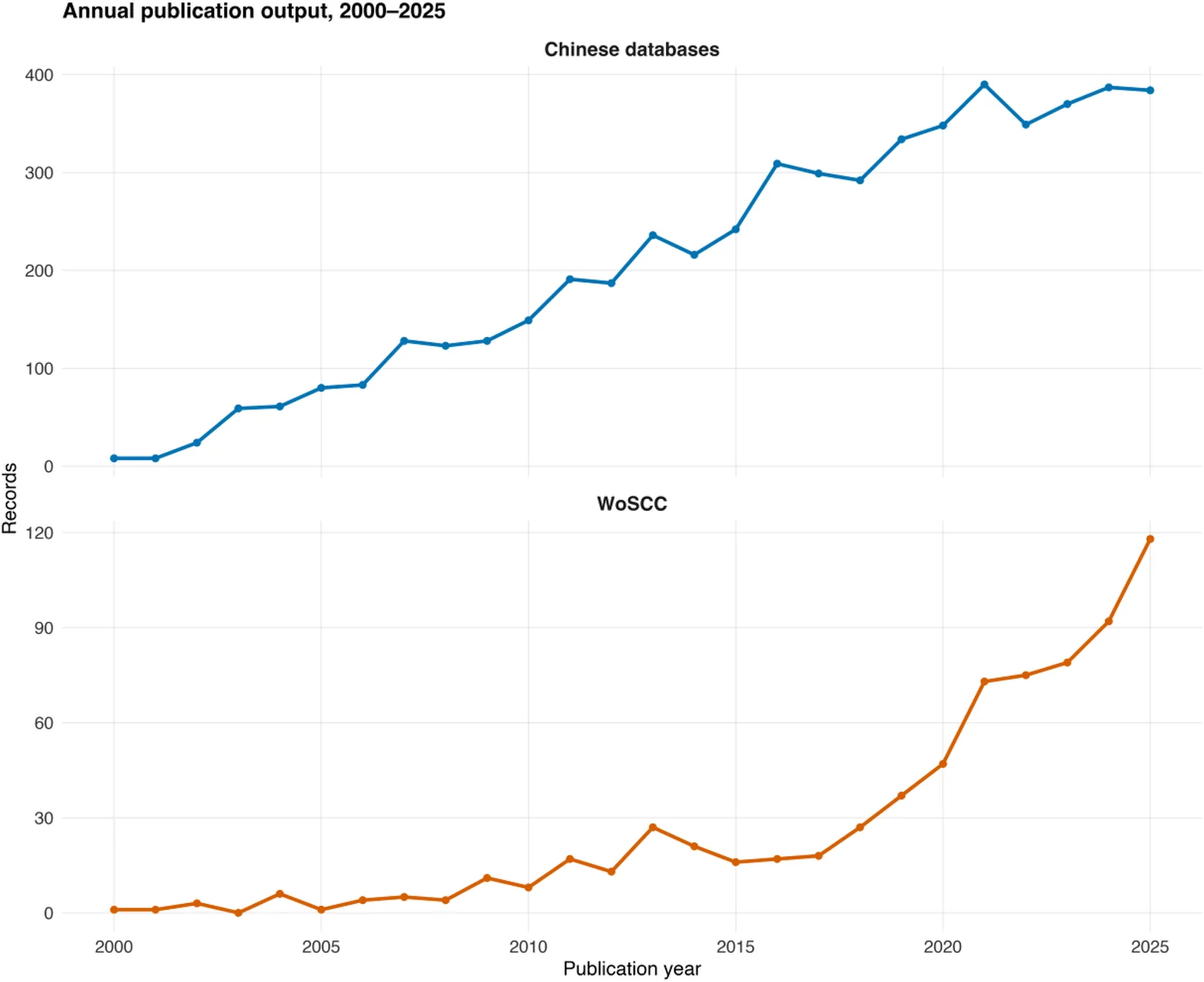
Annual publication counts in the Chinese and WoSCC corpora, 2000–2025.

### 3.3 Author collaboration

The complete Chinese author graph contained 12,141 nodes, 30,211 edges, density = 0.000410, and 2,128 components; the largest component included 5,882 nodes. The WoSCC graph contained 2,933 nodes, 15,360 edges, density = 0.003572, and 105 components; its largest component included 2,329 nodes. Tu Ya was the most frequent Chinese author (85 records), while Fu, WB led the WoSCC ranking (23). Frequency and betweenness describe labels within the retrieved graphs and do not resolve homonyms, establish individual identity, or measure quality. Author rankings are reported in Table 1, and collaboration networks are shown in Figure 4.

**Table 1:** Leading authors ranked by distinct-record frequency; display names do not merge analytical nodes.

Corpus	Rank	Author	*n*	Degree	Strength	Weighted betweenness
Chinese	1	Tu Ya	85	131	426	0.0624
Chinese	2	Fu Wenbin	73	157	371	0.0752
Chinese	3	Wang Lingling	46	40	128	0.0060
Chinese	4	Huang Yong	36	53	142	0.0094
Chinese	5	Lu Jun	36	65	173	0.0015
Chinese	6	Xu Shifen	35	49	113	0.0097
Chinese	7	Ren Lu	34	51	96	0.0159
Chinese	8	Li Zhigang	33	68	203	0.0193
Chinese	9	Wang Long	27	42	75	0.0118
Chinese	10	Wang Jian	26	66	89	0.0161
WoS	1	Fu, WB	23	121	207	0.1193
WoS	2	Wang, Y	22	129	172	0.0755
WoS	3	Xu, SF	17	62	120	0.0176
WoS	4	Huang, Y	16	83	131	0.0380
WoS	5	Wang, T	16	43	86	0.0315
WoS	6	Wang, L	15	114	126	0.0441
WoS	7	Liu, J	14	96	116	0.0476
WoS	8	Zhang, Y	14	106	109	0.0400
WoS	9	Li, Y	14	88	96	0.0321
WoS	10	Fang, JQ	14	64	126	0.0162

**Figure 4:**
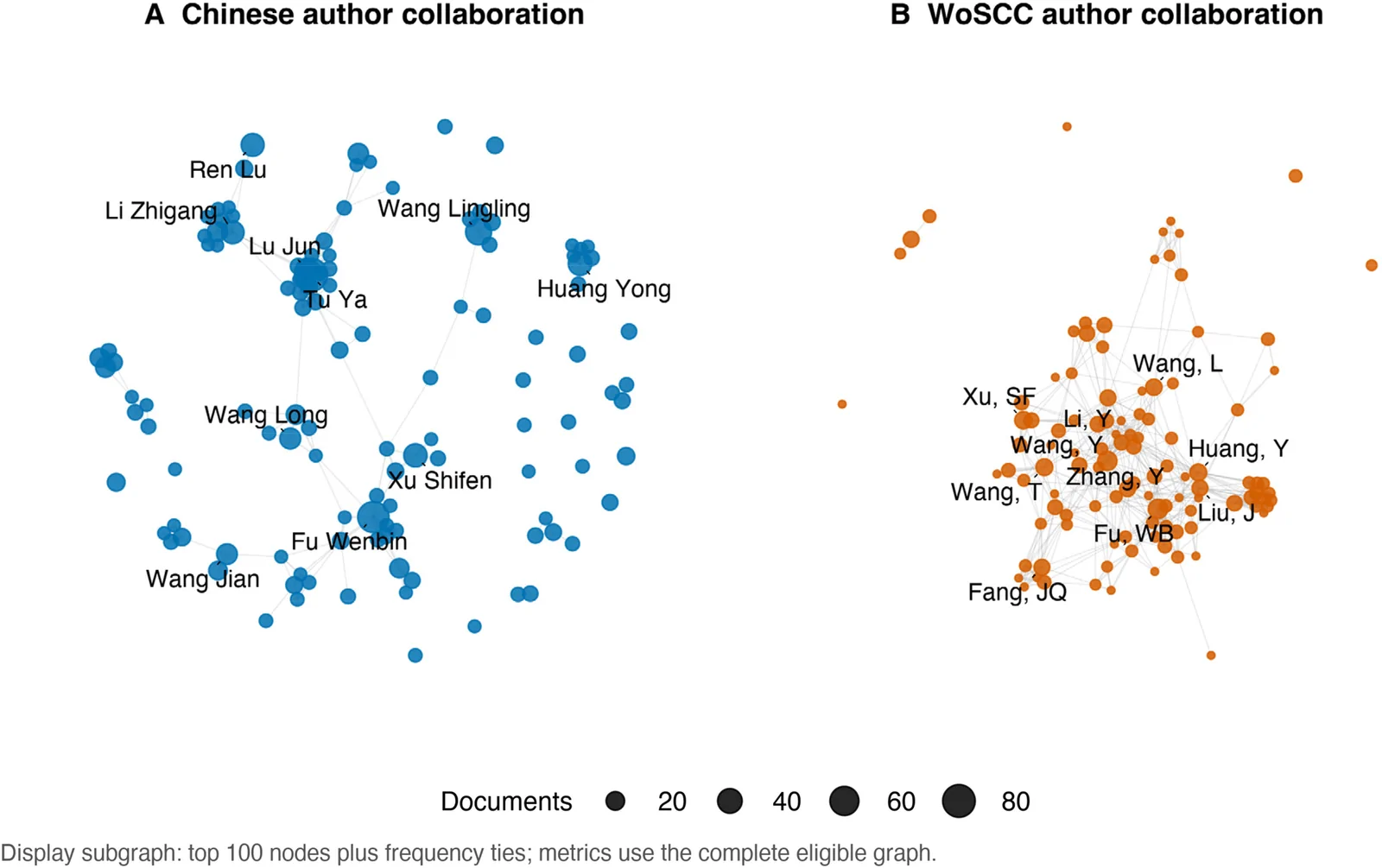
Author collaboration networks. Panel (**A**): Chinese author collaboration; Panel (**B**): WoSCC author collaboration. Display panels show the top 100 nodes plus frequency ties; metrics reported in the text and tables use complete graphs.

### 3.4 Institution and WoSCC country/region collaboration

The Chinese institution graph contained 5,810 nodes, 8,841 edges, density = 0.000524, and 1,577 components, with 2,647 nodes in the largest component. The WoSCC institution graph contained 491 nodes, 1,509 edges, density = 0.012544, and 56 components, with 376 nodes in the largest component. Beijing University of Chinese Medicine was the most frequent Chinese institution (238 records), while Guangzhou University of Chinese Medicine led the WoSCC institution ranking (72). Institution rankings remain sensitive to affiliation completeness and approved aliases. Institution rankings are reported in Table 2.

**Table 2:** Leading institutions ranked by distinct-record frequency; display names do not merge analytical nodes.

Corpus	Rank	Institution	*n*	Degree	Strength	Weighted betweenness
Chinese	1	Beijing University of Chinese Medicine	238	236	422	0.1025
Chinese	2	Heilongjiang University of Chinese Medicine	191	104	232	0.0238
Chinese	3	Tianjin University of Traditional Chinese Medicine	119	114	186	0.0290
Chinese	4	Guangzhou University of Chinese Medicine	109	148	236	0.0657
Chinese	5	First Teaching Hospital of Tianjin University of Traditional Chinese Medicine	104	51	124	0.0230
Chinese	6	Nanjing University of Chinese Medicine	92	85	110	0.0149
Chinese	7	First Affiliated Hospital of Heilongjiang University of Chinese Medicine	85	31	99	0.0079
Chinese	8	Chengdu University of Traditional Chinese Medicine	83	58	77	0.0095
Chinese	9	Liaoning University of Traditional Chinese Medicine	78	36	71	0.0076
Chinese	10	Shandong University of Traditional Chinese Medicine	71	62	128	0.0154
WoS	1	Guangzhou University of Chinese Medicine	72	65	133	0.2019
WoS	2	Shanghai University of Traditional Chinese Medicine	65	53	131	0.2291
WoS	3	Beijing University of Chinese Medicine	45	46	86	0.1231
WoS	4	Zhejiang Chinese Medical University	38	31	49	0.0356
WoS	5	University of Hong Kong	36	49	105	0.2311
WoS	6	China Academy of Chinese Medical Sciences	27	53	95	0.1096
WoS	7	Chengdu University of Traditional Chinese Medicine	27	30	40	0.0490
WoS	8	Harvard University	26	55	134	0.0729
WoS	9	Harvard University Medical Affiliates	22	42	113	0.0117
WoS	9	Massachusetts General Hospital	22	42	113	0.0117

Country/region analysis was restricted to WoSCC; the parser default in the other corpus was not treated as observed affiliation evidence. The WoSCC graph contained 32 nodes, 74 edges, density = 0.149194, and five components. China appeared in 570 WoSCC records, followed by the United States (91), Hong Kong (73), Australia (34), and South Korea (32). These counts reflect corpus coverage and coauthorship metadata, not comparative research quality. Source geographic labels were retained: China, Hong Kong, and Taiwan are separate database labels, without interpreting them as equivalent administrative units. These are full-counting affiliation frequencies; one record may contribute to several country/region labels, so their counts need not sum to 721. Country/region rankings are reported in Table 3; institution and country/region networks are shown in Figure 5.

**Table 3:** Leading WoSCC countries/regions ranked by distinct-record frequency.

Corpus	Rank	Country/region	*n*	Degree	Strength	Weighted betweenness
WoS	1	China	570	22	221	0.6484
WoS	2	United States	91	12	93	0.0559
WoS	3	Hong Kong	73	10	111	0.0000
WoS	4	Australia	34	9	39	0.0559
WoS	5	South Korea	32	8	26	0.0774
WoS	6	United Kingdom	31	10	25	0.0000
WoS	7	Taiwan	26	4	27	0.0000
WoS	8	Canada	16	9	27	0.0516
WoS	9	Malaysia	16	2	15	0.0000
WoS	10	Germany	15	13	22	0.1301

**Figure 5:**
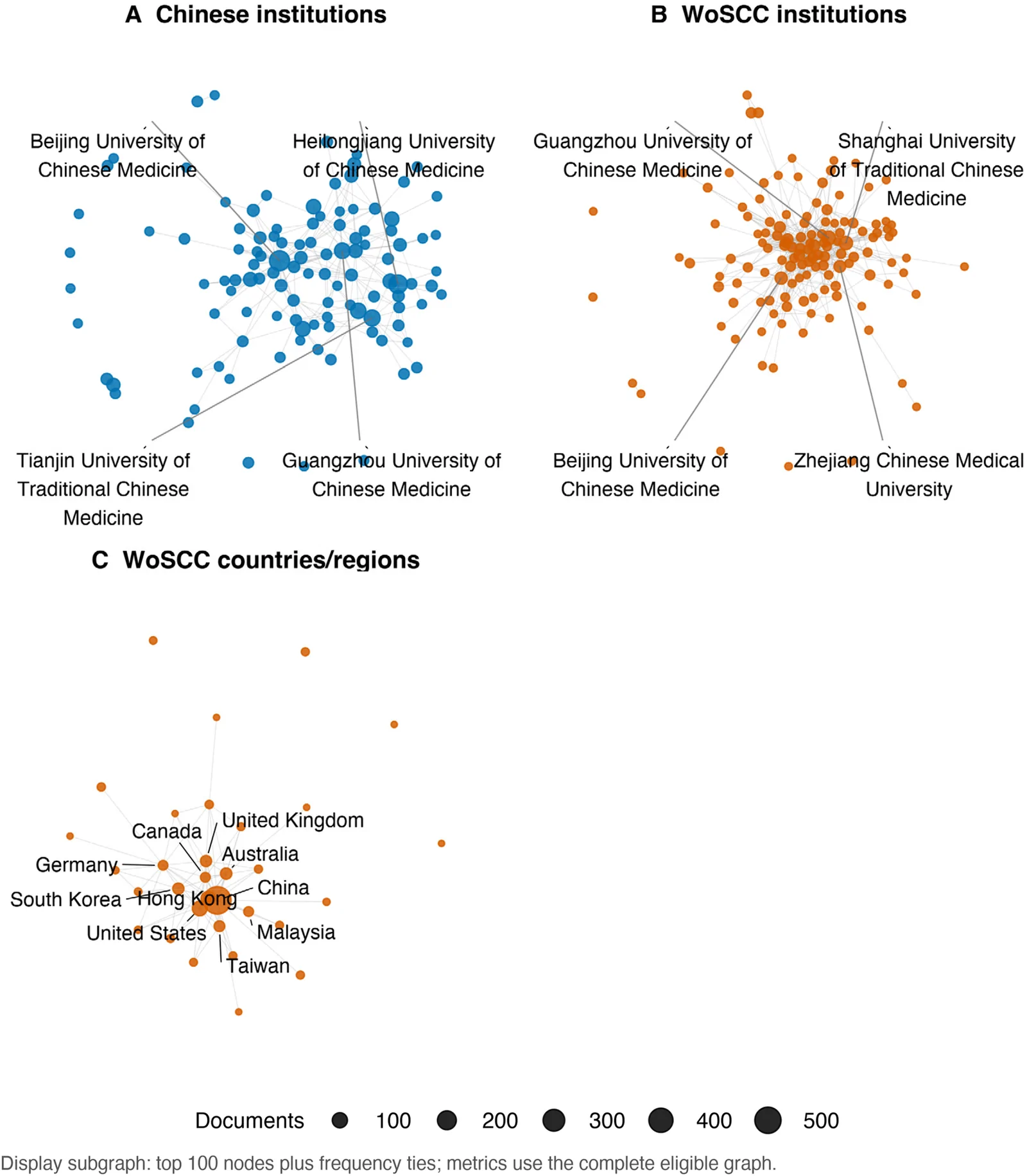
Institution collaboration in both corpora and country/region collaboration in WoSCC only. Panel (**A**): Chinese institutions; Panel (**B**): WoSCC institutions; Panel (**C**): WoSCC countries/regions. Display panels show the top 100 nodes plus frequency ties.

### 3.5 Keyword co-occurrence

At the five-record threshold, the Chinese keyword graph contained 569 nodes, 9,694 edges, density = 0.059989, and one component, while the WoSCC Author Keyword graph contained 81 nodes, 673 edges, density = 0.207716, and one component. Both were connected. The leading Chinese terms were Depressive disorder (1,919), Acupuncture (1,490), Acupuncture and moxibustion (1,028), Depression (1,009), and Poststroke depression (865). In WoSCC, the most frequent terms were Depression (355), Acupuncture (281), Electroacupuncture (104), Meta-analysis (84), and Anxiety (84). Keyword rankings are reported in Table 4, and co-occurrence networks are shown in Figure 6.

**Table 4:** Leading keywords ranked by distinct-record frequency; display names do not merge analytical nodes.

Corpus	Rank	Keyword	*n*	Degree	Strength	Weighted betweenness
Chinese	1	Depressive disorder	1,919	491	4,997	0.6856
Chinese	2	Acupuncture	1,490	469	4,107	0.5567
Chinese	3	Acupuncture and moxibustion	1,028	402	2,732	0.1496
Chinese	4	Depression	1,009	406	3,006	0.2826
Chinese	5	Poststroke depression	865	362	2,141	0.1467
Chinese	6	Electroacupuncture	649	315	1,808	0.0864
Chinese	7	Stroke	537	266	1,702	0.0282
Chinese	8	Anxiety	321	210	1,059	0.0047
Chinese	9	Review	244	143	732	0.0000
Chinese	10	Depressive disorder syndrome (Yuzheng)	162	183	458	0.0037
WoS	1	Depression	355	76	831	0.8082
WoS	2	Acupuncture	281	71	648	0.5176
WoS	3	Electroacupuncture	104	41	211	0.0498
WoS	4	Meta-analysis	84	41	266	0.0250
WoS	5	Anxiety	84	43	253	0.0005
WoS	6	Randomized controlled trial	69	45	199	0.0000
WoS	7	Poststroke depression	63	38	147	0.0244
WoS	8	Tai chi	59	38	153	0.0248
WoS	9	Systematic review	53	35	177	0.0000
WoS	10	Major depressive disorder	35	27	78	0.0000

**Figure 6:**
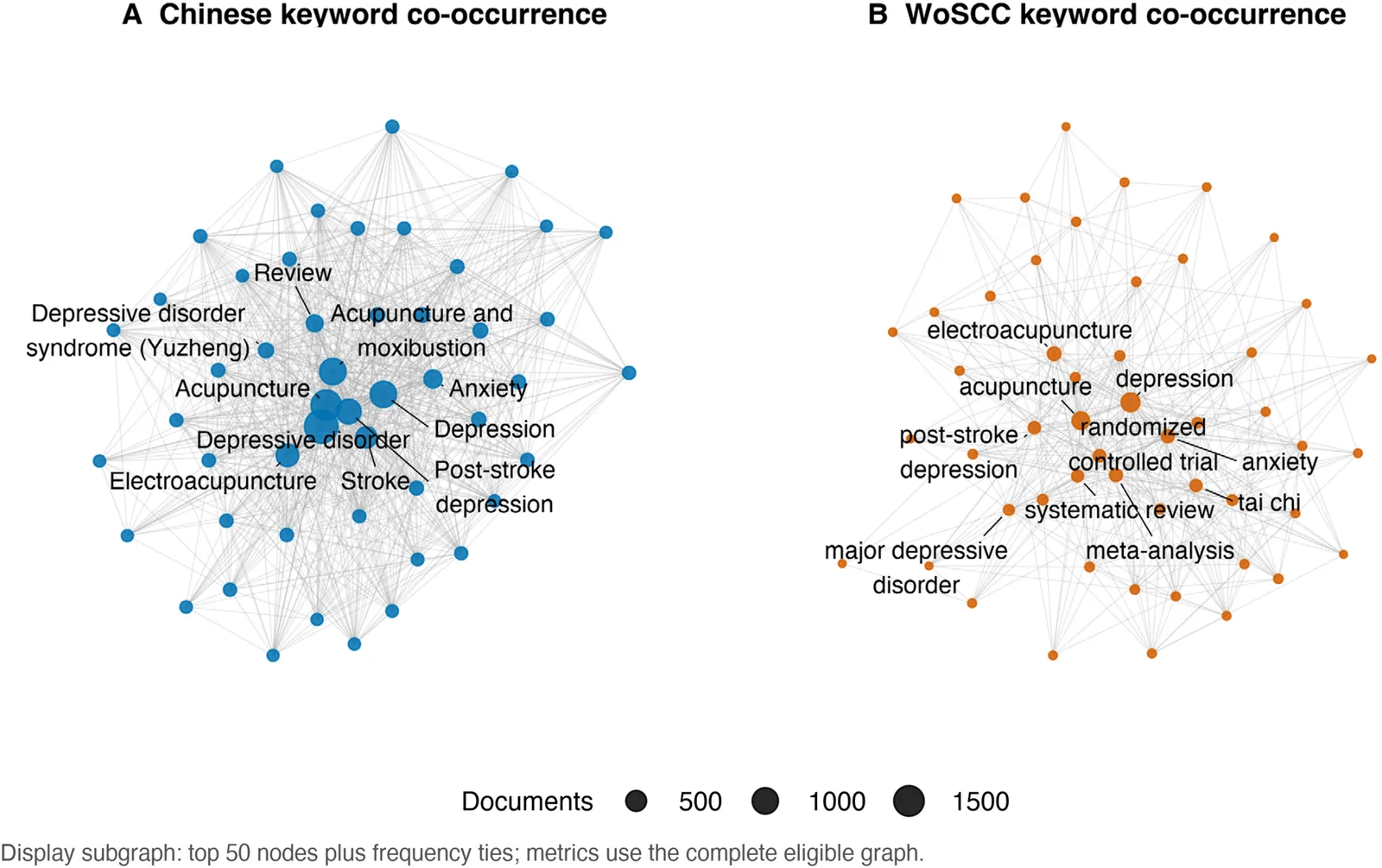
Keyword co-occurrence networks based on Chinese keywords and WoSCC author keywords. Panel (**A**): Chinese keyword co-occurrence; Panel (**B**): WoSCC keyword co-occurrence. The display subgraphs contain the top 50 nodes plus frequency ties; full analytical graphs remain machine-readable.

### 3.6 Exploratory keyword communities and stability

Weighted Louvain identified five Chinese communities (*Q* = 0.194124; mean cosine-distance silhouette = 0.005173) and four WoSCC communities (*Q* = 0.134767; mean silhouette=0.029369; Figure 7). Near-zero silhouettes indicate extensive overlap in record-use profiles and do not support sharply separated topic classes. Resolution 0.8, 1.0, and 1.2 produced 3, 5, and 11 Chinese communities and 2, 4, and 5 WoSCC communities.

**Figure 7:**
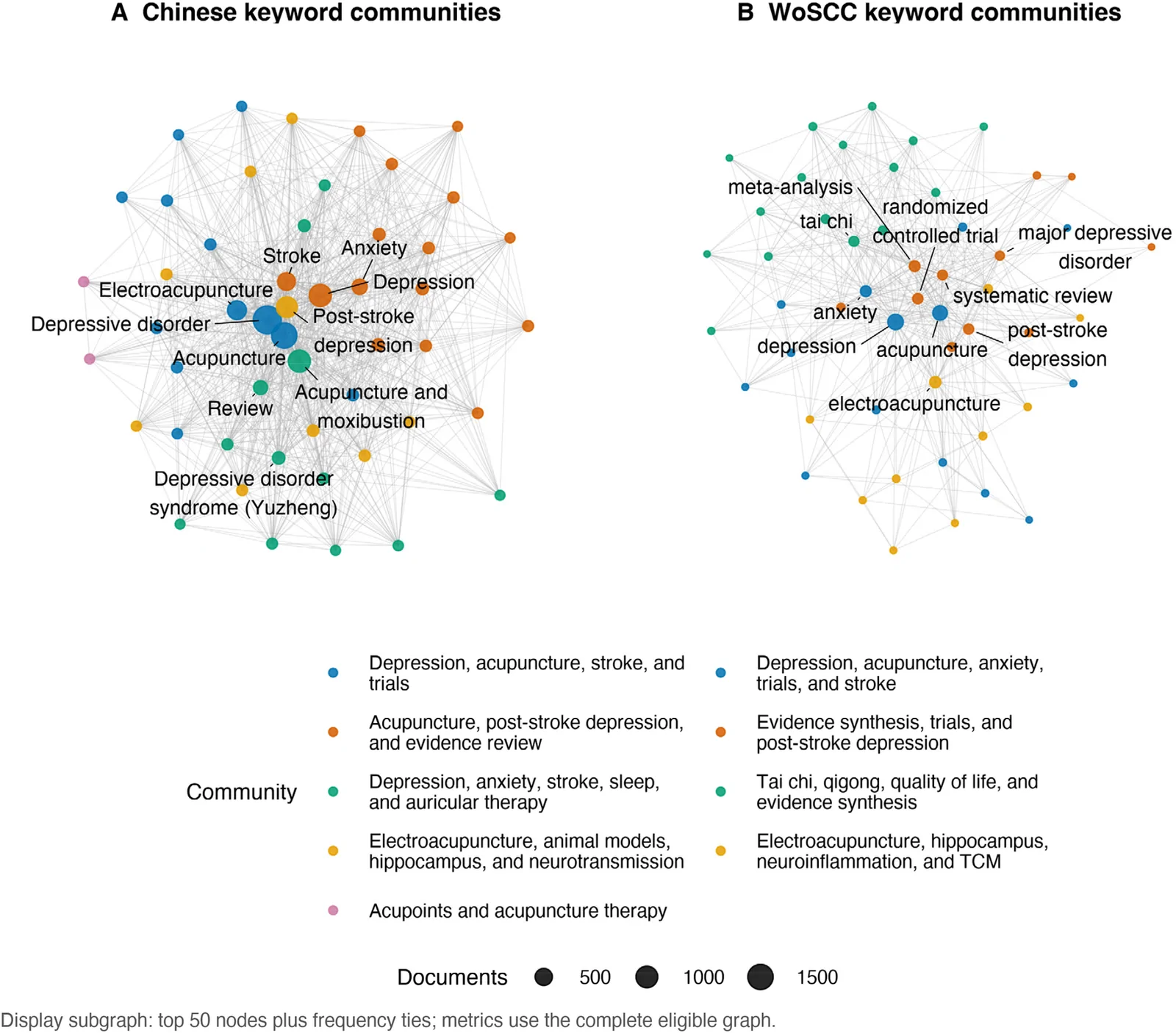
Exploratory weighted Louvain keyword communities. Panel (**A**): Chinese keyword communities; Panel (**B**): WoSCC keyword communities. Colors show the primary algorithmic partition. Near-zero silhouettes and sensitivity to resolution, seed, and algorithm indicate overlapping exploratory directions rather than discrete topic classes. Display subgraph: top 50 nodes plus frequency ties; metrics use the complete eligible graph.

Across 100 Louvain seeds, pairwise adjusted Rand indices had mean/median/minimum values of 0.544/0.521/0.313 for Chinese and 0.566/0.496/0.097 for WoSCC. Leiden produced 5 and 3 communities with adjusted Rand indices of 0.404 and 0.516 relative to primary Louvain. These findings reinforce the exploratory interpretation.

Merging broad depression terms reduced the Chinese keyword graph to 568 nodes and 9,327 edges (five communities; *Q* = 0.157583; silhouette = 0.005015) and the WoSCC graph to 80 nodes and 662 edges (three communities; *Q* = 0.142933; silhouette = 0.028599). Agreement with the primary partition among unchanged terms was ARI = 0.413 and 0.599, respectively. The merged concepts occurred in 2,918 Chinese and 364 WoSCC records. Weak separation persisted, while individual community membership remained sensitive to terminology (Supplementary Methods S4).

Across the expanded threshold-by-resolution grid, community counts ranged from 1 to 17 in Chinese and 1 to 12 in WoSCC. All six resolution-0.5 settings returned a single community; their silhouettes were undefined. In settings with multiple communities, silhouettes remained low (Chinese 0.0052–0.0086; WoSCC 0.0219–0.0418). Relative to the primary graph, strength rankings on shared nodes had Spearman correlations of 0.993–1.000 and 0.977–1.000, with 9–10 and 10 of the 10 highest-strength terms retained, respectively. Greedy modularity returned five communities in each corpus, with ARI = 0.585 and 0.568 against the primary Louvain. Stable prominence and unstable boundaries therefore support different levels of interpretation (Supplementary Methods S6).

The two linkage-sensitivity copies retained all 10 leading Chinese author, institution, and keyword labels by frequency. Annual counts differed by at most two records from the baseline, and the publication peak remained in 2021 (390 and 391 records). Author and institution graphs remained sparse. Keyword partitions contained five and six communities, with silhouettes of 0.00360 and 0.00414 and ARI = 0.538 and 0.951 against the baseline. Of the 112 original Chinese burst intervals, 108 and 109 retained the exact term and endpoints. These results support the tested descriptive rankings and sparse-network interpretation while showing that community assignment and individual burst boundaries remain conditional on linkage choices (Supplementary Methods S3).

Descriptive community labels were: Chinese 1: Depression, acupuncture, stroke, and trials; Chinese 2: Acupuncture, poststroke depression, and evidence review; Chinese 3: Depression, anxiety, stroke, sleep, and auricular therapy; Chinese 4: Electroacupuncture, animal models, hippocampus, and neurotransmission; Chinese 5: Acupoints and acupuncture therapy; WoSCC 1: Depression, acupuncture, anxiety, trials, and stroke; WoSCC 2: Evidence synthesis, trials, and poststroke depression; WoSCC 3: Tai chi, qigong, quality of life, and evidence synthesis; WoSCC 4: Electroacupuncture, hippocampus, neuroinflammation, and TCM. Labels summarize prominent terms and are neither clinical categories nor independent findings.

### 3.7 Burst intervals and whole-period community profiles

The strongest Chinese burst was Electroacupuncture (strength 59.49; 2000–2010). Later intervals included Sleep quality (19.90; 2022–2025) and Gut microbiota (9.93; 2024–2025). WoSCC produced three primary bursts: Protocol (4.90; 2020–2022), Neuroinflammation (4.23; 2024–2025), and Neural Regeneration (3.35; 2010–2015). Burst strength indicates temporal concentration within each corpus, not evidence of clinical importance.

Figure 8 uses independent scales by corpus because the number and magnitude of detectable bursts differed. Figure 9 reports annual prevalence of terms assigned to whole-period communities rather than communities re-estimated each year. Open circles mark years with fewer than 10 records; proportions based on small yearly denominators are unstable. Cross-corpus vertical differences should therefore not be read as effect contrasts.

**Figure 8:**
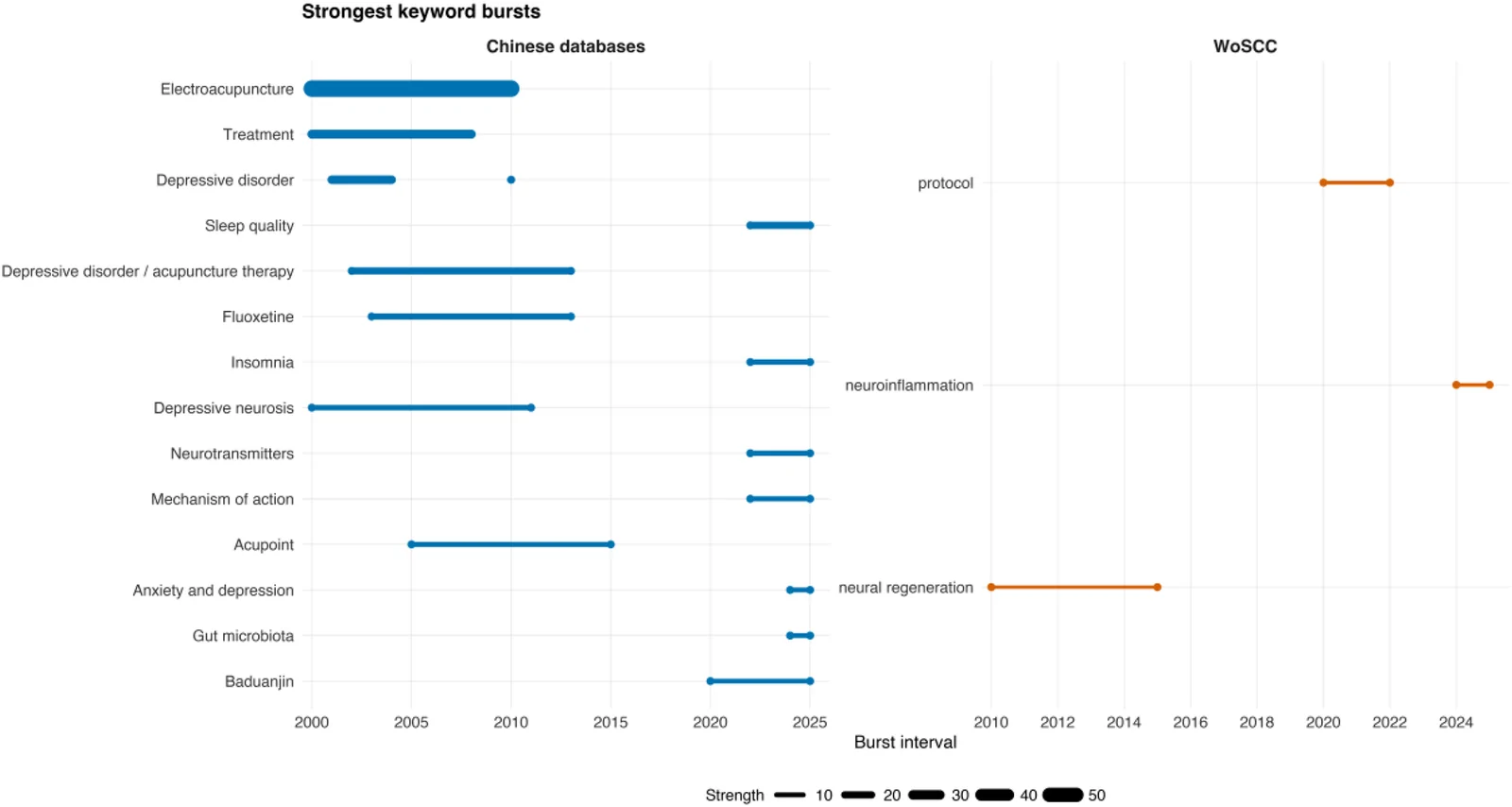
Strongest keyword burst intervals under the primary two-state specification (*s* = 2.0; gamma = 1.0). Facets use independent scales, and strength values are internal to this implementation.

**Figure 9:**
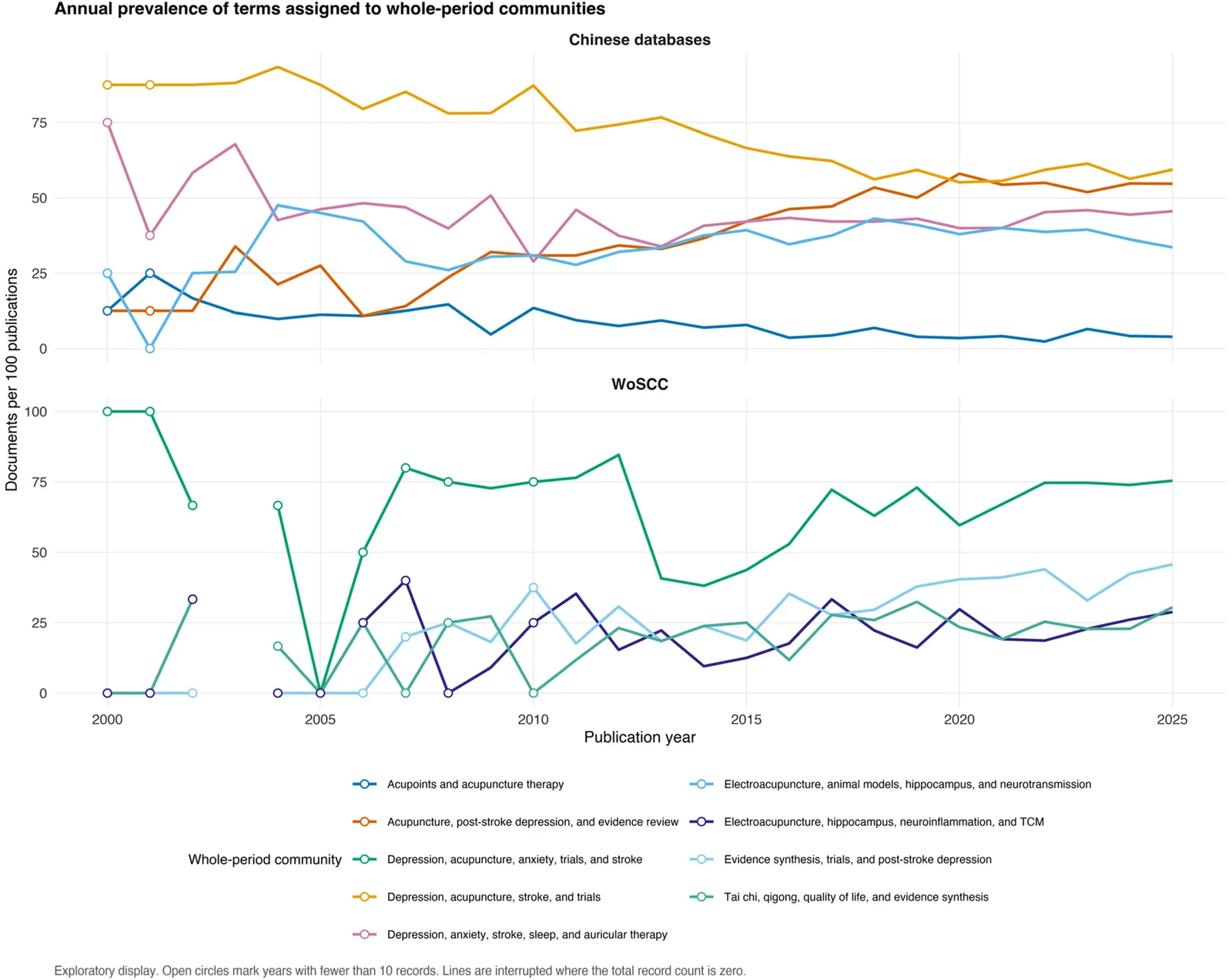
Annual prevalence of terms assigned to whole-period communities. Open circles indicate years with fewer than 10 records; years without records interrupt the lines. In WoSCC, 2003 has no records and undefined prevalence; 2005 has one record without Author Keywords, yielding zero observed prevalence under the all-record denominator but undefined prevalence under the available-keyword denominator. The denominator is all records in each year; keyword availability varied over time. Available-keyword denominator results in Supplementary Methods S8 materially change some WoSCC trajectories and peak years.

Author Keyword availability in WoSCC was 47.62% in 2014 and 96.74% in 2024. With available-keyword denominators, all three WoSCC primary burst terms and intervals remained, with strengths of 4.705, 3.850, and 3.552 for Protocol, Neuroinflammation, and Neural Regeneration, respectively. The Chinese burst count changed from 112 to 110, retaining 107 exact intervals. Community time profiles were more sensitive to denominators: WoSCC correlations between the two annual series ranged from 0.444 to 0.913, and differences reached 41.90 percentage points even in years with at least 10 records. The WoSCC evidence synthesis community peak shifted from 2025 to 2016. Chinese series correlations were 0.992–0.999, with unchanged peak-year sets. Figure 9 therefore describes observed keyword recording within the retained corpora and does not independently establish temporal growth in topic attention (Supplementary Methods S8; Supplementary Figure S1).

## 4 Discussion

### 4.1 Principal findings

This record-level analysis shows substantial growth in the literature and sparse collaboration, especially in the larger Chinese author and institution graphs. Acupuncture-related terminology dominated both corpora. Poststroke depression, evidence synthesis, traditional exercise, sleep, and experimental terms recurred, but the community solutions were weakly separated and only moderately stable. The appropriate interpretation is therefore a map of overlapping research attention rather than a taxonomy of distinct clinical domains.

### 4.2 Relation to prior bibliometric and clinical evidence

The prominence of acupuncture, depression, sleep, and mechanistic terms overlaps with Kong et al. (5), whereas evidence synthesis, trial, poststroke depression, and experimental themes overlap with Song et al. (6). Condition-specific maps provide narrower acupuncture-focused comparators (7–9). Sun et al. is the closest recent comparison because it also combined Chinese and English sources over 2000–2025, but it focused on acupuncture and used CNKI, Wanfang, WoSCC, and PubMed (10). The present contribution is therefore not the first cross-language coverage; it encompasses a broader non-pharmacological TCM scope, inclusion of VIP, separate corpus-specific analysis, and an auditable normalization and validation design.

The 5,385-record Chinese and 721-record WoSCC corpora are not directly comparable with the datasets reported in prior studies. Database coverage, dates, fields, document types, intervention definitions, screening, and normalization can all change counts and networks. Clinical syntheses of acupuncture and mind-body exercise provide a separate evidence context (11, 12), while current guideline-based care places treatment choices within shared decision-making and established psychological, pharmacological, and structured activity options (13). Neither those external clinical sources nor the present bibliometric patterns justify inferring efficacy from publication prominence.

The contribution tests which descriptive patterns endure wider intervention coverage and explicit analytical choices. Acupuncture remained prominent in the primary keyword rankings, whereas the broad-term merger altered community assignments without producing well-separated communities. The expanded grid retained most high-strength terms but sometimes merged all keywords into one community. Thus, the evidence supports sustained attention to acupuncture alongside other overlapping directions, rather than an invariant partition of the field. High-frequency term survival after filtering is partly guaranteed by the selection rule and is not an independent validation; the recalculated strength ranks and alternative partitions provide additional diagnostic evidence. Differences in coverage cannot be disentangled from differences in underlying scientific priorities with this design.

### 4.3 Collaboration and thematic interpretation

Low densities and many components indicate that collaboration was distributed across numerous teams. The WoSCC graphs were smaller and denser; these properties co-occurred with different levels of affiliation completeness and indexing scope. Their individual contributions cannot be separated by the present descriptive comparison. Frequency and weighted betweenness identify structurally prominent labels within the retained graphs but do not establish quality, field-normalized productivity, independence, or clinical impact.

Recent bursts for sleep quality, gut microbiota, and neuroinflammation indicate when terminology became concentrated. Such signals can motivate targeted clinical or mechanistic appraisal, but they do not demonstrate therapeutic benefit or causal pathways. Community membership also changed materially under different resolution, random seed, and Leiden sensitivity. Reporting those diagnostics prevents visually distinct colors from being mistaken for stable scientific categories. The keyword-denominator analysis further separates the timing of the three retained WoSCC burst signals from broader community trajectories: the former retained their intervals, whereas several community peaks moved. Community trajectories should not be described as sustained growth or emergence without accounting for time-varying keyword availability.

### 4.4 Strengths and limitations

Strengths include explicit record-level Chinese deduplication, a rule-defined boundary queue with documented decision provenance, a fixed publication-year eligibility window, separate corpora, logged mappings, complete graph exports, fixed parameters, resolution and burst sensitivity, a 100-seed stability analysis, Leiden sensitivity, and an R/igraph cross-check of selected graph quantities. Publication tables preserve both source and display labels, so translation does not alter analytical identity.

Limitations include database- and language-dependent coverage, non-equivalent metadata structures, and source scopes that preclude direct cross-corpus comparisons. SinoMed was not searched. The WoSCC interface used Index Date, and record-level publication-year exclusions cannot recover eligible records omitted by that retrieval setting. Chinese retrieval also left three displayed records without parseable exports. Paired assessments covered the rule-flagged queue; unflagged decisions were not exhaustively double-screened. The duplicate audit sampled logged candidate pairs and cannot measure missed duplicates among all possible record pairs. Its reference assessment identified errors in both candidate strata. Record identities were made mutually consistent across the entire sampled pair set, and constrained linkage copies evaluated selected downstream consequences. The primary corpus remains the descriptive baseline; these sensitivity copies do not estimate the complete literature or resolve unsampled identity errors. Source-level eligibility was held fixed, and forward/reverse processing of unreviewed historical links is a limited-order check rather than an exhaustive identity uncertainty analysis. Betweenness and all alternative-algorithm settings were not re-estimated in the linkage copies. Shared records may induce dependence between sampled pairs, limiting the nominal coverage of the descriptive Wilson intervals. Author Keywords were missing for 99 WoSCC records, and institution data were absent for 37 WoSCC and 40 Chinese records. Chinese records lacked cited-reference lists. Author homonyms and initials were not disambiguated, and institution rankings depend on approved aliases. The original run environment was incompletely recorded, so later verification does not reconstruct its historical identity. The corpus mixes articles and reviews, and record counts do not represent independent experiments or patients. Technique-based eligibility also allowed medicated acupoint preparations and combined regimens; this design cannot isolate the non-pharmacological contribution to any treatment effect. Near-zero silhouettes and sensitivity to resolution, seed, algorithm, and synonym handling limit claims about individual communities. Burst results depend on annual denominators and model settings. Available-keyword denominators preserved the three WoSCC burst intervals but materially changed several community profiles; conditioning on available keywords does not eliminate non-random missingness. Community memberships and individual burst intervals are therefore not uniformly robust across the checks performed.

## 5 Conclusion

From 2000 through 2025, the literature on non-pharmacological traditional Chinese medicine interventions for depression expanded across the included Chinese databases and WoSCC. The bibliographic landscape centered on acupuncture-related interventions and included poststroke depression, evidence synthesis terminology, traditional exercises, sleep, and experimental themes. Collaboration networks were sparse, and thematic interpretations and document coverage overlapped across communities. Temporal community profiles were sensitive to keyword availability and should be interpreted as recorded metadata patterns. These findings provide a reproducible descriptive map of the retained records; efficacy, safety, mechanisms, and causal claims require separate study-level evidence.

## Data Availability

The original contributions presented in the study are included in the article/Supplementary Material, further inquiries can be directed to the corresponding author. Licensed raw database exports are not redistributed because access and reuse are governed by the originating databases. The reproducibility materials accompanying the manuscript include code, mappings, derived tables, and cross-check records, together with machine-readable graph files, run metadata, and environment locks. No public repository identifier is claimed. Supplementary materials are available from the corresponding author upon reasonable request.
