# Supplementary material for: Non-pharmacological traditional Chinese medicine interventions for depression: bibliometric mapping of Chinese databases and WoSCC, 2000–2025
Source: Front Med (Lausanne). 2026 Oct 8;13:1964791. doi: 10.3389/fmed.2026.1964791 (PMC13687034; doi:10.3389/fmed.2026.1964791)
Supplement: Supplementary file 1 [file Table1.docx]

**Bibliometric Reporting Checklist**

This checklist maps bibliometric reporting items to the manuscript sections and Supplementary Methods S1–S10.

## BIBLIO crosswalk

The following grouped crosswalk covers the BIBLIO minimum reporting requirements (manuscript reference 15); related items are grouped for readability.

| **Area** | **Reporting requirement** | **Location** |
| --- | --- | --- |
| Title | Title identifies bibliometric mapping | Manuscript title |
| Abstract | Structured objective, methods, results and conclusion | Abstract |
| Background | Context and rationale | Introduction |
| Objectives | Central question and aims | Introduction final paragraphs |
| Sources | Databases and scope | Methods 2.1; S1 |
| Search | Expressions and settings | Methods 2.2; S1 |
| Period | 2000–2025 and its rationale | Methods 2.1 |
| Eligibility | Intervention and record criteria | Methods 2.3; S2 |
| Refinement | Deduplication and screening | Methods 2.3; Results 3.1; S2, S3 |
| Quality assessment | Process QA; no clinical risk-of-bias synthesis | Methods 2.1, 2.7; S3, S6, S8, S9 |
| Data handling | Normalization and analysis | Methods 2.4–2.7; S3, S4, S5, S6, S7, S8, S9 |
| Flow | Selection totals | Results 3.1; Figure 2; S2 |
| Findings | Publication and entity summaries | Results 3.2–3.5; Tables 1–4 |
| Maps | Community, burst and temporal results | Results 3.6–3.7; Figures 4–9 |
| Synthesis | Prominence and unstable boundaries | Discussion 4.1–4.3 |
| Limitations | Coverage, linkage and parameter limits | Discussion 4.4; S3, S4, S6, S8 |
| Conclusion | Descriptive scope and evidence limits | Conclusion |

## PRISMA S crosswalk

| **Item** | **Reporting status** | **Location** |
| --- | --- | --- |
| 1 Databases and platforms | CNKI, Wanfang, VIP, WoSCC; editions listed | Methods 2.1; S1 |
| 2 Multiple databases | Separate native expressions and fields | S1.1–S1.4 |
| 3 Study registries | Not searched | Methods 2.1 defines database-only scope |
| 4 Online resources | No supplementary online-source search | Methods 2.1 defines database-only scope |
| 5 Citation searching | Not used for corpus retrieval | Methods 2.1; citation-analysis scope in S10 |
| 6 Contacts | No contact-based retrieval reported | Database-only source scope |
| 7 Other methods | No additional retrieval method reported | Methods 2.1 |
| 8 Full strategies | Chinese expressions and WoSCC topic expression | S1 |
| 9 Limits | Date, language and publication-type settings | Methods 2.1–2.2; S1 |
| 10 Search filters | No named validated methodological filter claimed | S1 |
| 11 Prior work | No adapted published search strategy claimed | S1 |
| 12 Updates | Exports supplemented; no new database rerun claimed | Methods 2.1; S1 |
| 13 Dates | Search, export and publication eligibility distinguished | S1 |
| 14 Peer review | No formal PRESS review claimed | Not performed within the reported workflow |
| 15 Records | Displayed, exported and included totals distinguished | S1; Results 3.1; Figure 2 |
| 16 Deduplication | Rules, identity consistency and linkage sensitivity separated | S3 |
