# Supplementary material for: Non-pharmacological traditional Chinese medicine interventions for depression: bibliometric mapping of Chinese databases and WoSCC, 2000–2025
Source: Front Med (Lausanne). 2026 Oct 8;13:1964791. doi: 10.3389/fmed.2026.1964791 (PMC13687034; doi:10.3389/fmed.2026.1964791)
Supplement: Supplementary file 2 [file Table2.docx]

**Supplementary Methods**

## S1. Database-specific searches

Searches covered journal literature published from 2000 through 2025. Chinese database searches were performed on 15 July 2026; exports were collected and supplemented through 17 July 2026. The retained WoSCC export was downloaded on 17 July 2026. The search dates, fields, expressions, interface settings, and export denominators are specified below. The eligibility window refers to publication year, which is distinct from the WoSCC retrieval interface date field.

### S1.1 CNKI

TI denotes title, KY keywords, and TKA title, keywords, and abstract.

(TI = ('抑郁' + '抑郁症' + '抑郁障碍' + '郁证') OR KY = ('抑郁' + '抑郁症' + '抑郁障碍' + '郁证')) AND TKA = ('针刺' + '针灸' + '电针' + '头针' + '耳针' + '艾灸' + '灸法' + '穴位贴敷' + '穴位敷贴' + '耳穴贴压' + '耳穴压豆' + '耳穴埋豆' + '耳穴埋籽' + '拔罐' + '刮痧' + '推拿' + '气功' + '太极拳' + '八段锦' + '五禽戏' + '六字诀' + '导引' + '五行音乐' + '中医音乐' + '中医适宜技术')

The search logs indicate Academic Journals, all journals, and 2000–2025. Bilingual expansion was enabled and synonym expansion was disabled. The logged results count was 4,758 records; 4,757 records were successfully exported and parsed. No additional Chinese-language filter was applied.

### S1.2 Wanfang

The query used the title-or-keyword field and the topic field. The complete export was collected on 17 July 2026.

题名或关键词:(抑郁 OR 抑郁症 OR 抑郁障碍 OR 郁证) AND 主题:(针刺 OR 针灸 OR 电针 OR 头针 OR 耳针 OR 艾灸 OR 灸法 OR 穴位贴敷 OR 穴位敷贴 OR 耳穴贴压 OR 耳穴压豆 OR 耳穴埋豆 OR 耳穴埋籽 OR 拔罐 OR 刮痧 OR 推拿 OR 气功 OR 太极拳 OR 八段锦 OR 五禽戏 OR 六字诀 OR 导引 OR 五行音乐 OR 中医音乐 OR 中医适宜技术)

The search logs indicate journal articles, 1 January 2000 through 31 December 2025, and 5,568 results. Bilingual and subject-term expansion were disabled. All 5,568 records were exported and parsed. No additional Chinese-language filter was applied.

### S1.3 VIP

T denotes title, K keywords, and M topic.

(T=抑郁 OR T=抑郁症 OR T=抑郁障碍 OR T=郁证 OR K=抑郁 OR K=抑郁症 OR K=抑郁障碍 OR K=郁证) AND (M=针刺 OR M=针灸 OR M=电针 OR M=头针 OR M=耳针 OR M=艾灸 OR M=灸法 OR M=穴位贴敷 OR M=穴位敷贴 OR M=耳穴贴压 OR M=耳穴压豆 OR M=耳穴埋豆 OR M=耳穴埋籽 OR M=拔罐 OR M=刮痧 OR M=推拿 OR M=气功 OR M=太极拳 OR M=八段锦 OR M=五禽戏 OR M=六字诀 OR M=导引 OR M=五行音乐 OR M=中医音乐 OR M=中医适宜技术)

The search logs indicate all journals, all disciplines, 2000–2025, and 4,645 results. A total of 4,643 records were successfully exported and parsed. No additional Chinese-language filter was applied.

### S1.4 Web of Science Core Collection

The database was Web of Science Core Collection, using SCI-EXPANDED, SSCI, and ESCI. The complete topic expression was:

TS=(depress* OR dysthymi* OR "depressive disorder" OR "depressive disorders")
AND TS=(acupunctur* OR electroacupunctur* OR "electro-acupuncture"
OR "scalp acupuncture" OR "auricular acupuncture" OR moxibust*
OR "acupoint application" OR "acupoint sticking" OR "acupoint plaster*"
OR "auricular acupressure" OR "ear acupressure" OR "ear seed*"
OR cupping OR "gua sha" OR guasha OR tuina OR "tui na"
OR qigong OR "qi gong" OR "tai chi" OR taichi OR taiji*
OR baduanjin OR "ba duan jin" OR wuqinxi OR liuzijue OR daoyin
OR "five-element music" OR "five element music"
OR "traditional Chinese music" OR "TCM music"
OR "appropriate TCM technolog*" OR "appropriate technolog* of traditional Chinese medicine")

TS searched title, abstract, Author Keywords, and Keywords Plus. Exact Search was off; quoted phrases and explicit truncation were retained as written. Interface refinements were English and Article or Review Article. The interface Index Date range was 1 January 2000 to 31 December 2025. The search and export date was 17 July 2026. The retained export comprised 1,260 unique accession numbers in three batches; this export count is reported separately from the query specification. Publication eligibility was assessed subsequently from each record’s PY field; the Index Date filter was not described as a Publication Year search filter.

Twelve records had PY=2026 despite 2025 Early Access dates. They were outside the publication-year window; exclusion categories in the final flow used a mutually exclusive reason hierarchy, so not every year-ineligible record appears under the year category. Record-level filtering cannot recover eligible publications omitted by the interface date restriction. The three Chinese sources showed 14,971 results but supplied 14,968 parseable records; the three missing exports were not imputed as bibliographic records.

Chinese searches used native Chinese expressions and journal-only settings; eligibility retained Chinese-language records. No English-only expression replaced a Chinese query. Database-native fields are not interchangeable across platforms.

## S2. Eligibility and screening

### S2.1 Eligibility criteria and intervention categories

Eligibility protocol TX-01 version 1.0 defines the following intervention categories and eligibility hierarchy. It is an eligibility framework, distinct from search vocabulary and from communities discovered by network analysis. Categories are nonexclusive; a record may receive more than one intervention code but contributes only once to corpus counts. The review was not registered, and no prospective registration is claimed.

The intervention categories are listed in Table S1.

Table S1. Intervention categories and included techniques.

| **Code** | **Category** | **Included techniques** |
| --- | --- | --- |
| T1 | Needling | Manual, electro-, scalp, and auricular acupuncture |
| T2 | Thermal stimulation | Moxibustion and acupoint-directed moxa techniques |
| T3 | Non-invasive acupoint techniques | Acupoint application, ear-seed pressing, auricular acupressure |
| T4 | Manual and surface techniques | Tuina, cupping, gua sha |
| T5 | Movement and breathing practices | Qigong, tai chi, Baduanjin, Wuqinxi, Liuzijue, daoyin |
| T6 | TCM-based music | Five-element music and explicitly TCM-based music therapy |
| T7 | Identifiable combinations | Combinations containing at least one separately identifiable T1–T6 technique |

Depression and an eligible intervention had to be a primary topic in title or keywords. Generic relaxation, general exercise, ordinary massage, non-TCM music, pharmacological TCM alone, and an incidental abstract mention were insufficient. Broad labels such as appropriate TCM technology required a named T1–T6 component. When title or keyword wording was ambiguous, the abstract clarified the described technique; it did not convert an incidental mention into a primary-topic record. Acupoint products were included only as an identifiable acupoint application technique; systemic herbal medication alone was excluded. Preparations used at acupoints may have pharmacological actions. This operational technique category does not establish that the intervention is pharmacologically inert. Adjunctive medication did not exclude an otherwise eligible named technique. Mixed interventions required the eligible component to be identifiable as an analyzed topic, without claiming that its clinical effect could be isolated.

Depression encompassed depressive disorders, depression symptoms, and depression in defined clinical contexts, including post-stroke depression. Laboratory records required an explicit depression-related topic and eligible technique. Ineligible publication type and year were evaluated alongside topic eligibility. Missing abstract or keyword fields alone did not exclude a record with adequate primary-topic evidence. For the TX-01 examples, the priority was insufficient identifying evidence, ineligible document type, ineligible year, a confirmed duplicate of the same article, absent primary depression topic, no identifiable eligible technique, and an incidental intervention mention. Corpus selection and the worked examples use separately documented exclusion hierarchies. Exclusions used one final reason per record; the selection table retained other flags to prevent mutually exclusive flow totals from being mistaken for all overlapping reasons.

### S2.2 Worked eligibility examples

The worked examples illustrate the application of eligibility protocol TX-01 version 1.0. They are separate from the corpus and the 2,089-record paired-assessment matrix and do not estimate screening accuracy.

Table S2 presents the worked eligibility examples.

Table S2. Worked eligibility examples and decision rules.

| **Example** | **Decision** | **Applied rule** |
| --- | --- | --- |
| Depression and manual, electro-, scalp or auricular acupuncture as primary topics | Include | T1 |
| Depression and moxibustion | Include | T2 |
| Ear-seed pressing or identifiable acupoint application | Include | T3; medicated preparations do not imply pharmacological inactivity |
| Depression and tuina, cupping or gua sha | Include | T4 |
| Qigong, tai chi, Baduanjin, Wuqinxi, Liuzijue or daoyin | Include | T5; depression must remain a primary topic |
| Five-element or explicitly TCM-based music | Include | T6 |
| Acupuncture plus qigong, or an eligible technique adjunctive to medication | Include | Identifiable component; T7 where multiple techniques are combined |
| Broad appropriate-TCM-technology label without a named technique | Exclude | E6; broad vocabulary alone is insufficient |
| Ordinary exercise, massage, non-TCM music or systemic herbal treatment alone | Exclude | E6; no eligible technique established |
| Depression or the intervention mentioned only incidentally | Exclude | E5 or E7 |
| Missing abstract or keywords with an informative title and adequate identifiers | Include | Missing fields alone do not override adequate topic evidence |
| Depression-model laboratory study with an eligible technique | Include | Experimental context is permitted |
| Related report confirmed to be a different article | Include | Bibliographic identity differs; theme overlap is not duplication |
| Same-article duplicate version, out-of-window year or ineligible type | Exclude | E4, E3 or E2, subject to the stated priority |

The example log reports initial decisions, uncertainty or disagreement, adjudication, final reasons, and the applicable protocol version.

### S2.3 Boundary screening and reconciliation

Non-empty review_flags defined the boundary queue. Flags represented ambiguous depression relevance, technique only in an abstract, unspecified intervention, incomplete essential metadata, year or publication-type anomalies, and related record-version issues. The queue contained 1,529 Chinese and 560 WoSCC records. Unflagged records followed the same eligibility hierarchy without mandatory paired assessment; they were not described as independently double-screened.

Two reviewers completed Include, Exclude, or Uncertain assessments without access to the other’s initial decision. Agreement statistics were calculated before reconciliation. The three-category matrix was:

Table S3 reports the initial decision matrix.

Table S3. Initial paired-screening decision matrix.

| **Reviewer A / Reviewer B** | **Include** | **Exclude** | **Uncertain** |
| --- | --- | --- | --- |
| Include | 739 | 121 | 56 |
| Exclude | 89 | 913 | 58 |
| Uncertain | 43 | 64 | 6 |

Observed agreement was 1,658/2,089=79.37%. Cohen’s kappa was 0.623, using all three decision categories and their marginal frequencies. There were 431 unequal decision pairs and six pairs in which both reviewers selected Uncertain. All 437 required reconciliation. In addition, comparison with the fixed reference decisions corrected 4 concordant classifications. The reference decisions and final eligibility rules governed the final table; reconciliation was not an additional independent accuracy assessment. No Uncertain final decision remained.

Table S4 reports the final counts by screening route.

Table S4. Final inclusion and exclusion counts by screening route.

| **Route** | **Chinese included** | **Chinese excluded** | **WoSCC included** | **WoSCC excluded** |
| --- | --- | --- | --- | --- |
| Boundary queue | 794 | 735 | 137 | 423 |
| Unflagged rules | 4591 | 143 | 584 | 116 |
| Total | 5385 | 878 | 721 | 539 |

## S3. Deduplication and reference assessment

### S3.1 Matching and representative records

Chinese records were linked only when traceable evidence met one of three rules:

1. exact normalized DOI;
2. exact normalized title of at least six characters, years within one year, and at least one of overlapping authors, matching first author, or matching normalized journal;
3. normalized-title similarity of at least 0.96 under Python difflib.SequenceMatcher, together with the same compatibility rule as route 2. Candidate generation required normalized titles of at least 12 characters, an identical first eight characters, and a shared year-neighborhood bucket. The final compatibility check still required publication years within one year.

Generic same-title records with incompatible author, journal, or year evidence were retained as distinct. Exact DOI linkage did not impose an additional title-similarity threshold, allowing different language displays of the same DOI. Links were consolidated transitively with union-find; supporting link counts therefore differ from duplicate-record deletions. The retained-distinct candidate log contains exact-title pairs failing compatibility; it is not a log of every possible rejected fuzzy pair. The representative record was selected using field completeness, number of authors, abstract length, and a source priority of CNKI, Wanfang, then VIP; all source identifiers were retained. Keywords and institutions were unioned with provenance-preserving normalization.

### S3.2 Initial consistency review

The 14,968 Chinese exports were consolidated to 6,263 records, removing 8,705 duplicate source records. The audit contains 13,788 supporting merge pairs and 1,028 retained-distinct candidate pairs; pair counts are not deletion counts. A random sample of 200 merge pairs and 200 retained-distinct candidate pairs was assessed independently by two reviewers. Sampling was without replacement within each pair-log stratum. Reviewers compared normalized DOI, title, authors, journal, and publication year. Differences were reconciled against the fixed reference decision and matching hierarchy; 14 merge-pair and 16 retained-distinct-pair assessments disagreed, and one additional concordant retained-distinct assessment required reference correction. No unresolved review decisions remained. This initial stage is a consistency audit against the original decisions, not an independent estimate of over-merging or missed-duplicate rates. The same pairs then received the separate reference assessment described below; the stages and outcomes are not interchangeable. Sampling candidate pairs cannot measure recall among unflagged pairs; pair-level results must not be extrapolated to all removed source records.

### S3.3 Reference assessment

The second-stage assessment used the same 200 merged and 200 retained-distinct pairs sampled in the initial consistency review. Algorithm decisions, linkage-rule labels, and the initial consistency-review results were concealed from two reference reviewers. They evaluated whether the two records represented the same bibliographic article using title, authors, journal, year, and DOI together. Distinct related reports were retained separately; an identifier conflict prompted examination of the remaining fields rather than unconditional acceptance of a shared DOI. A third reviewer adjudicated disagreements and completed a final reference check. Final identity did not default to the algorithm output. This approach follows methodological work separating reference-based false-positive and false-negative duplicate decisions; it is not a benchmark of those papers’ software products (manuscript references 16–17). Same-article initial reference links were represented as an undirected graph. Its connected components defined record identities; final pair labels were determined by identity equality. This applies reflexivity, symmetry, and transitivity across all sampled records. Initial decisions remain separately recorded, and any pair changed by component closure is reported rather than silently overwritten.

Table S5 reports the reference classifications and stratum-specific error proportions.

Table S5. Reference assessment of sampled duplicate candidates.

| **Algorithm stratum** | **Reference duplicate** | **Reference distinct** | **Error proportion** | **95% Wilson interval** |
| --- | --- | --- | --- | --- |
| Merged pairs | 195 | 5 | 5/200 = 2.50% | 1.07–5.72% |
| Retained-distinct candidate pairs | 20 | 180 | 20/200 = 10.00% | 6.57–14.94% |

There were 10 initial reference-review disagreements in the merged stratum and 8 in the retained-distinct stratum. Record-identity consistency corrected one concordant initial classification in the retained-distinct stratum. After this correction, all 400 final pair decisions agreed with the record-identity components, with no remaining same/different contradiction. These 18 disagreements differ from the 30 disagreements in the initial consistency review. False merges comprised three related but distinct reports and two same-title different articles. Missed candidate duplicates comprised eight year conflicts, six cases with missing metadata, five author variants, and one additional identity-consistency link. The last category is a logical reconciliation category, not an observed bibliographic defect. Error-type counts describe the final reference discrepancy; they do not attribute each error to a proven software defect.

For an error count k among n=200 sampled pairs, the Wilson interval used z=1.959964, p=k/n, center=(p+z²/(2n))/(1+z²/n), and half-width=z*sqrt(p*(1-p)/n+z²/(4n²))/(1+z²/n). Intervals treat pairs as independent and are descriptive; overlapping source records and the restricted candidate frame limit generalization. The equal stratum samples were not pooled because their sampling fractions differ. The retained-distinct error proportion is conditional on candidate rejection, not the fraction of all true duplicates missed. No claim is made about unflagged pairs, unsampled linkage components, or a population-wide sensitivity/specificity. The primary 5,385/721-record corpus was retained as the descriptive baseline. The linkage copies below implement reference relationships at the record level; the pair counts themselves cannot simply be subtracted from or added to corpus totals.

### S3.4 Linkage sensitivity

The analysis evaluated how the sampled identity discrepancies could affect the descriptive results. It used the same 13,393 Chinese source records represented by the 5,385 baseline records, holding source-level inclusion fixed. Final same-article constraints from the reference assessment were applied first. Historical automatic merge links were then processed in lexicographic source-ID order; a link was rejected if joining its components would violate any final different-article constraint. Reverse lexicographic order supplied a second check because identities of unsampled links were not independently established. Both complete partitions satisfied all 400 sampled reference constraints before restriction to the fixed included source set. Excluded sources supplied linkage context only and did not add bibliographic content or become newly eligible observations.

Unchanged components retained their baseline records. Changed components were rebuilt from their included source members using the original representative rule: bibliographic-field completeness, author count, abstract length, and CNKI/Wanfang/VIP priority. Authors came from the most informative source list; institutions and keywords were unioned with within-record uniqueness. Source members were neither fabricated nor deleted, but their grouping changed in the sensitivity copies. Publication year followed the representative record. All resulting years remained within 2000–2025. The copies are sensitivity analyses, not corrected estimates of total eligible literature; eligibility was not independently re-screened after splitting or joining components.

Table S6 summarizes record counts and annual changes in the linkage copies.

Table S6. Record counts and annual changes in linkage-sensitivity copies.

| **Copy** | **Records** | **Changed output records** | **Rejected historical links** | **2021 records** | **Max annual change** |
| --- | --- | --- | --- | --- | --- |
| forward | 5386 | 37 | 40 | 390 | 2 |
| reverse | 5387 | 38 | 39 | 391 | 2 |

Rejected links are edge-level events, not counts of deleted articles. Changed-output counts include split or joined components and are not net record differences. Both orders preserved all 13,393 source members exactly once. The record-count changes were +1 and +2, and the annual peak remained in 2021.

Table S7 reports the corresponding network summaries.

Table S7. Network summaries in linkage-sensitivity copies.

| **Copy** | **Entity** | **Nodes** | **Edges** | **Density** | **Components** |
| --- | --- | --- | --- | --- | --- |
| primary | author | 12141 | 30211 | 0.000410 | 2128 |
| primary | institution | 5810 | 8841 | 0.000524 | 1577 |
| primary | keyword | 569 | 9694 | 0.059989 | 1 |
| forward | author | 12126 | 30187 | 0.000411 | 2125 |
| forward | institution | 5810 | 8896 | 0.000527 | 1568 |
| forward | keyword | 570 | 9727 | 0.059982 | 1 |
| reverse | author | 12130 | 30194 | 0.000410 | 2124 |
| reverse | institution | 5810 | 8907 | 0.000528 | 1568 |
| reverse | keyword | 569 | 9711 | 0.060094 | 1 |

Table S8 reports the corresponding keyword community results.

Table S8. Keyword community results in linkage-sensitivity copies.

| **Copy** | **Keyword communities** | **Q** | **Silhouette** | **ARI vs baseline** |
| --- | --- | --- | --- | --- |
| primary | 5 | 0.194124 | 0.005173 | 1.000 |
| forward | 5 | 0.195426 | 0.003603 | 0.538 |
| reverse | 6 | 0.194214 | 0.004137 | 0.951 |

All ten leading author, institution, and keyword labels by frequency, and all ten by strength, were retained in each copy. These compare sets with canonical-label tie-breaking, not identical rank order. Keyword-frequency rank correlations were 0.99968 and 0.99956; strength-rank correlations were 0.99951 and 0.99932 on shared nodes. Maximum keyword-frequency differences were three and four records. Author frequency changed by at most one; institution frequency changed by at most two and three. The sparse collaboration and broad keyword-prominence descriptions persisted, while community membership remained conditional on linkage. Weighted betweenness was not recalculated, so its ranking stability is not claimed.

Both copies yielded 112 Chinese burst intervals. Exact term/start/end agreement retained 108 and 109 of the baseline 112; the rest changed or were replaced. The complete interval files retain these differences. These checks do not establish stability of every burst, every centrality, all unreviewed duplicate pairs, or all alternative community algorithms. WoSCC was unchanged because this duplicate-linkage assessment concerned the Chinese sources.

## S4. Metadata and entity normalization

### S4.1 Metadata availability and comparison scope

Table S9 summarizes metadata availability in each corpus.

Table S9. Metadata availability by corpus.

| **Corpus** | **Field** | **Present** | **Total** | **Completeness** |
| --- | --- | --- | --- | --- |
| Chinese | Authors | 5380 | 5385 | 99.91% |
| Chinese | Institutions | 5345 | 5385 | 99.26% |
| Chinese | Keywords | 5330 | 5385 | 98.98% |
| WoSCC | Authors | 721 | 721 | 100.00% |
| WoSCC | Institutions | 684 | 721 | 94.87% |
| WoSCC | Countries/regions | 717 | 721 | 99.45% |
| WoSCC | Author Keywords | 622 | 721 | 86.27% |

Chinese country values generated by a parser default were not treated as observed country evidence. Author Keywords and Keywords Plus were not pooled in the WoSCC primary analysis. The two corpora differ in coverage, document mix, field definitions, metadata completeness, and size. Counts, densities, keyword ranks, and communities are interpreted within each corpus; no inferential comparison identifies a language or national effect. Country/region labels follow the WoSCC affiliation export and are not equal administrative units. China, Hong Kong, and Taiwan remain distinct source labels. Full counting permits a record to contribute to multiple labels; geographic frequencies are not an exclusive partition of 721 records. Bibliometric measures describe attention and structure, not intervention efficacy, safety, mechanisms, or clinical recommendations.

### S4.2 Entity normalization and mappings

Mechanical normalization applied Unicode NFKC, whitespace collapse, and removal of surrounding punctuation. Keyword and WoSCC country matching also used case normalization. Within each record, a canonical entity contributed at most once. Only mapping rows marked approved changed analytical identity; pending or retained-distinct rows did not merge nodes. English publication labels were display translations and did not change analytical nodes.

Version 3 mappings cover Chinese keywords, WoSCC keywords, institutions, countries/regions, and publication display labels. Their file identities are retained in the accompanying technical record (Technical_File_Identities.tsv).

In the primary analysis, the source terms 抑郁 and 抑郁症 remained separate analytical nodes and were displayed as Depression and Depressive disorder. This conservative non-merging rule should not be interpreted as proof that the concepts are clinically non-equivalent. Cross-language display labels do not establish complete conceptual equivalence. Abbreviations or spelling variants were merged only through a recorded approved mapping; case, Unicode, spacing, and surrounding punctuation were standardized mechanically. Major depressive disorder and post-stroke depression retained their narrower meanings.

### S4.3 Terminology sensitivity

The primary mapping retained broad source terms separately. The alternative mapping combined 抑郁 with 抑郁症 in Chinese and depression with depressive disorder in WoSCC. Each record contributed at most once to the combined concept. More specific conditions, including major depressive disorder and post-stroke depression, remained distinct. Both mappings used frequency at least five, full counting, Louvain resolution 1.0, seed 20260803, and cosine-distance silhouettes on binary record profiles.

Table S10 compares the primary and alternative terminology mappings.

Table S10. Keyword network and community results under alternative terminology mappings.

| **Corpus** | **Mapping** | **Nodes** | **Edges** | **Communities** | **Q** | **Silhouette** |
| --- | --- | --- | --- | --- | --- | --- |
| chinese | retained | 569 | 9694 | 5 | 0.194124 | 0.005173 |
| chinese | merged | 568 | 9327 | 5 | 0.157583 | 0.005015 |
| wos | retained | 81 | 673 | 4 | 0.134767 | 0.029369 |
| wos | merged | 80 | 662 | 3 | 0.142933 | 0.028599 |

The Chinese concepts occurred in 1,919 and 1,009 records, with 10 records containing both, giving a union of 2,918. The WoSCC concepts occurred in 355 and 9 records, with no overlap, giving a union of 364. ARI between primary and merged partitions was computed only on unchanged terms, excluding the original and combined labels: 0.413 for Chinese and 0.599 for WoSCC. The primary and alternative matrices were constructed anew from record-level occurrences; edges were not combined by adding aggregate weights because that could double-count co-occurring synonyms. The Chinese community count remained five, while the WoSCC count changed from four to three. Silhouettes remained near zero. Therefore broad terminology choices alter membership, and interpretation should emphasize overlapping directions rather than fixed community labels.

## S5. Network construction and visualization

Full counting was used. Each record contributed once to every non-empty entity and once to every unordered within-record pair. Node frequency was distinct-record frequency; degree was the number of adjacent nodes; strength was the sum of edge weights; normalized weighted betweenness used inverse co-occurrence weight as distance.

Author, institution, and WoSCC country/region analytical graphs retained all non-empty entities. Keyword graphs retained terms occurring in at least five distinct records. Reported metrics were calculated on these complete eligible graphs.

Figures 4 and 5 used a display-only induced subgraph based on the top 100 nodes ranked by frequency descending, weighted betweenness descending, and canonical label ascending, with all nodes tied at the frequency cutoff retained. Figures 6 and 7 used the same rule with a top-50 cutoff. Actual displayed nodes were 118/109 for Chinese/WoSCC authors, 112/130 for Chinese/WoSCC institutions, 32 for WoSCC countries/regions, and 50/51 for Chinese/WoSCC keywords. Institution panels labelled the top four nodes; other panels labelled the top ten. Fruchterman-Reingold layouts used deterministic panel-specific seeds and 1,000 iterations. Network panels were exported at a final width of 6.5 inches. Labels used collision-aware displacement with connecting segments while preserving node positions, selected entities, edges, frequencies, and communities. Node-label text was 2.6 mm (approximately 7.4 points) at final size. Display-subgraph metrics were not substituted for complete-graph metrics.

## S6. Community detection and sensitivity

### S6.1 Primary partition and local stability

The primary weighted Louvain analysis used resolution 1.0 and seed 20260803. Communities were ordered by aggregate document frequency and given descriptive labels based on prominent terms. Separation was assessed from binary record-by-keyword profiles using cosine-distance silhouettes. Each keyword has one algorithmic community assignment. A document can contain terms from several communities, and community interpretations can overlap; neither circumstance represents multiple community assignments for a keyword.

Resolution sensitivity used 0.8, 1.0, and 1.2. Seed stability used 100 seeds from 20260803 through 20260902 and summarized adjusted Rand indices against the primary partition and among all seed pairs. R/igraph Leiden partitions supplied an alternative-algorithm sensitivity analysis. Near-zero silhouettes and material variation across resolution, seed, and algorithm require community labels to be interpreted as overlapping exploratory directions, not stable clinical or scientific classes.

### S6.2 Parameter rationale

The primary five-record support threshold removes extremely infrequent terms while retaining less common directions; it is a pragmatic analytical choice rather than a clinically validated cutoff. Thresholds of three and ten test a less restrictive and a more restrictive rule. Resolution 1.0 corresponds to conventional modularity; values below and above one favor larger and smaller communities, respectively. The broader 0.5/1.0/1.5 grid complements the original local 0.8/1.0/1.2 analysis and deliberately includes settings that may over-aggregate or fragment the field. All combinations used seed 20260803 and the existing source mappings. The expanded grid evaluates sensitivity to the keyword threshold and community resolution. No setting was chosen because it maximized separation or supported a preferred interpretation.

The full threshold-by-resolution results follow. Silhouette is NA for a single community because between-community separation is undefined, not zero. ARI compares the partition with primary Louvain only on shared nodes. Modularity at different resolutions optimizes different objectives; complete Q and edge-count outputs are retained in the machine-readable parameter table and are not ranked across resolutions to select a preferred setting.

### S6.3 Chinese parameter grid

Table S11 reports the Chinese parameter grid.

Table S11. Chinese keyword threshold-by-resolution results.

| **Minimum frequency** | **Resolution** | **Nodes** | **Communities** | **Silhouette** | **ARI on shared nodes** |
| --- | --- | --- | --- | --- | --- |
| 3 | 0.5 | 1044 | 1 | NA | 0.000 |
| 3 | 1.0 | 1044 | 10 | 0.006328 | 0.544 |
| 3 | 1.5 | 1044 | 17 | 0.007786 | 0.297 |
| 5 | 0.5 | 569 | 1 | NA | 0.000 |
| 5 | 1.0 | 569 | 5 | 0.005173 | 1.000 |
| 5 | 1.5 | 569 | 16 | 0.007483 | 0.412 |
| 10 | 0.5 | 279 | 1 | NA | 0.000 |
| 10 | 1.0 | 279 | 5 | 0.006674 | 0.524 |
| 10 | 1.5 | 279 | 14 | 0.008607 | 0.431 |

### S6.4 WoSCC parameter grid

Table S12 reports the WoSCC parameter grid.

Table S12. WoSCC keyword threshold-by-resolution results.

| **Minimum frequency** | **Resolution** | **Nodes** | **Communities** | **Silhouette** | **ARI on shared nodes** |
| --- | --- | --- | --- | --- | --- |
| 3 | 0.5 | 184 | 1 | NA | 0.000 |
| 3 | 1.0 | 184 | 4 | 0.021950 | 0.401 |
| 3 | 1.5 | 184 | 12 | 0.040084 | 0.418 |
| 5 | 0.5 | 81 | 1 | NA | 0.000 |
| 5 | 1.0 | 81 | 4 | 0.029369 | 1.000 |
| 5 | 1.5 | 81 | 7 | 0.035130 | 0.447 |
| 10 | 0.5 | 34 | 1 | NA | 0.000 |
| 10 | 1.0 | 34 | 3 | 0.041829 | 0.713 |
| 10 | 1.5 | 34 | 7 | 0.029413 | 0.504 |

### S6.5 Alternative algorithm and interpretation

At the primary frequency threshold, weighted greedy modularity used agglomerative community merging, providing a different optimization route from Louvain’s local moves and aggregation (manuscript reference 22). It used resolution 1.0 on the same complete graph; no visualization filter was applied. This is an algorithmic comparison, not an executed CiteSpace or VOSviewer platform benchmark. The earlier R/igraph Leiden results remain a separate cross-engine comparison.

Table S13 reports the greedy-modularity comparison.

Table S13. Greedy-modularity community results.

| **Corpus** | **Communities** | **Q** | **Silhouette** | **ARI versus Louvain** |
| --- | --- | --- | --- | --- |
| chinese | 5 | 0.196261 | 0.003579 | 0.585 |
| wos | 5 | 0.119871 | 0.031096 | 0.568 |

Strength was recalculated from each threshold-specific induced graph, then ranked on nodes shared with the primary graph using Spearman correlation with average ranks for ties. The correlation ranges were 0.993–1.000 in Chinese and 0.977–1.000 in WoSCC. The ten highest-strength terms shared 9–10 Chinese and 10 WoSCC terms with the primary graph, using canonical labels to break strength ties. These ranges include the unchanged primary setting. Repeating a resolution at the same frequency threshold does not change strength; this is not counted as independent rank evidence. High-frequency survival alone is expected under a frequency filter and is not an unbiasedness test.

All resolution-0.5 partitions collapsed to a single community. Multicommunity solutions had silhouettes of 0.0052–0.0086 in Chinese and 0.0219–0.0418 in WoSCC; greedy-modularity silhouettes were similarly low. Taken together with the earlier Leiden and terminology results, the checks support descriptive prominence within a fixed corpus but not stable community boundaries or an absence of bias. Publication totals and full author/institution graphs are unaffected by keyword parameters; this sensitivity analysis therefore does not provide additional validation of those quantities.

## S7. Burst detection

For a term with annual frequency k_t and annual corpus exposure n_t, the low-state probability was p0 = sum(k_t) / sum(n_t). The high-state probability was p1 = min(s * p0, 1 - epsilon). For state j in year t, the emission cost was the negative log-likelihood under a binomial model:

-log[choose(n_t, k_t) * p_j^k_t * (1 - p_j)^(n_t - k_t)]

A deterministic dynamic program selected the minimum-cost sequence over two states. Probabilities were clipped at epsilon=10^(-12) and 1-epsilon for likelihood evaluation. At the first year, the low-state cost was its emission cost and the high-state cost was its emission cost plus the entry penalty. Moving from high to low or remaining in either state had no transition penalty. Ties selected the low predecessor (including entry rather than stay for a high-state tie), and a terminal tie selected the low state. Backtracking recovered the path. A year with zero exposure contributed zero emission cost but remained in the 26-year sequence. Only positive finite-strength intervals were retained; strength excluded the transition penalty. Entering the high state incurred a transition penalty of gamma * log(T), where T=26 years. Contiguous high-state years defined an interval. Strength was the sum within that interval of low-state emission cost minus high-state emission cost.

The primary specification used s=2.0, gamma=1.0, and total frequency at least five. Sensitivity crossed s=1.5, 2.0, 2.5 with gamma=0.5, 1.0, 2.0. The Chinese primary analysis returned 112 intervals representing 108 unique terms; the WoSCC primary analysis returned three intervals. Among the 20 highest-strength Chinese primary intervals, the associated keyword was detected in all nine parameter settings for 14 intervals and in seven or eight settings for the other six; detection did not require identical interval endpoints. The WoSCC primary terms Protocol, Neuroinflammation, and Neural Regeneration were detected in six, five, and five of nine settings, respectively. Burst strength is internal to this two-state adaptation and is not comparable with CiteSpace burst strength. R/igraph did not independently recompute burst intervals.

The baseline s=2 doubles the term probability subject to its cap, while gamma=1 sets one log(T) entry penalty. The sensitivity grid varies probability contrast and entry cost; it is not an optimized fit or a reproduction of CiteSpace defaults.

## S8. Temporal denominators and keyword completeness

The primary annual prevalence is the number of records containing at least one term from a whole-period community divided by all records in that year. The alternative denominator counts records with non-empty keywords (Chinese keywords or WoSCC Author Keywords). Community membership, numerators, yearly axis, and primary burst settings s=2 and gamma=1 were held constant. A record can contribute to more than one community, so community percentages need not sum to 100%. If no records have keywords, conditional prevalence is unavailable rather than zero. If the total record count is zero, both proportions are unavailable and the lines are interrupted. The complete 26-year grid is retained, including these missing values. For the burst calculation, such a year has zero exposure and zero observed events within the complete 26-year sequence; it supplies no emission evidence. Neither approach recovers the unobserved keywords or resolves missingness related to journal, publication type, or year.

### S8.1 Annual keyword completeness

Table S14 reports annual keyword completeness.

Table S14. Annual keyword availability and completeness.

| **Year** | **Chinese available/all** | **Chinese %** | **WoSCC available/all** | **WoSCC %** |
| --- | --- | --- | --- | --- |
| 2000 | 8/8 | 100.00 | 1/1 | 100.00 |
| 2001 | 8/8 | 100.00 | 1/1 | 100.00 |
| 2002 | 24/24 | 100.00 | 2/3 | 66.67 |
| 2003 | 56/59 | 94.92 | 0/0 | NA |
| 2004 | 61/61 | 100.00 | 4/6 | 66.67 |
| 2005 | 80/80 | 100.00 | 0/1 | 0.00 |
| 2006 | 83/83 | 100.00 | 2/4 | 50.00 |
| 2007 | 128/128 | 100.00 | 5/5 | 100.00 |
| 2008 | 122/123 | 99.19 | 3/4 | 75.00 |
| 2009 | 126/128 | 98.44 | 8/11 | 72.73 |
| 2010 | 146/149 | 97.99 | 6/8 | 75.00 |
| 2011 | 186/191 | 97.38 | 15/17 | 88.24 |
| 2012 | 182/187 | 97.33 | 11/13 | 84.62 |
| 2013 | 231/236 | 97.88 | 15/27 | 55.56 |
| 2014 | 216/216 | 100.00 | 10/21 | 47.62 |
| 2015 | 240/242 | 99.17 | 11/16 | 68.75 |
| 2016 | 308/309 | 99.68 | 11/17 | 64.71 |
| 2017 | 297/299 | 99.33 | 15/18 | 83.33 |
| 2018 | 292/292 | 100.00 | 24/27 | 88.89 |
| 2019 | 332/334 | 99.40 | 31/37 | 83.78 |
| 2020 | 342/348 | 98.28 | 40/47 | 85.11 |
| 2021 | 386/390 | 98.97 | 65/73 | 89.04 |
| 2022 | 348/349 | 99.71 | 68/75 | 90.67 |
| 2023 | 365/370 | 98.65 | 73/79 | 92.41 |
| 2024 | 384/387 | 99.22 | 89/92 | 96.74 |
| 2025 | 379/384 | 98.70 | 112/118 | 94.92 |

WoSCC had no records in 2003 and one record without Author Keywords in 2005. Conditional community prevalence is unavailable in both years. The 2013 and 2014 WoSCC completeness values were 55.56% and 47.62%, compared with 96.74% in 2024 and 94.92% in 2025. The overall 86.27% figure therefore obscures substantial annual differences.

### S8.2 Burst comparison

Table S15 compares burst-interval retention between the two denominators.

Table S15. Burst-interval retention under alternative annual denominators.

| **Corpus** | **All-record intervals** | **Available-keyword intervals** | **Exact intervals retained** |
| --- | --- | --- | --- |
| Chinese | 112 | 110 | 107 |
| WoSCC | 3 | 3 | 3 |

Table S16 compares WoSCC burst strengths between the two denominators.

Table S16. WoSCC burst strengths under alternative annual denominators.

| **WoSCC term** | **Interval** | **All-record strength** | **Available-keyword strength** |
| --- | --- | --- | --- |
| neural regeneration | 2010–2015 | 3.350 | 3.552 |
| neuroinflammation | 2024–2025 | 4.228 | 3.850 |
| protocol | 2020–2022 | 4.899 | 4.705 |

In Chinese, the intervals for 临床观察 and 抑郁障碍 shortened by one year; HAMD评分, 效果, and 针刺 no longer had their original intervals, while 针灸 acquired a 2002–2003 interval. The full outer-joined comparison is retained in the machine-readable output. Preserving the three WoSCC burst intervals does not establish robustness of all Chinese bursts or of broader community trajectories.

### S8.3 Community annual profiles

Table S17 summarizes the differences between community time profiles.

Table S17. Community time-profile differences under alternative annual denominators.

| **Corpus/community** | **Year-series rho** | **Max change pp** | **Year** | **Max change pp with n≥10** |
| --- | --- | --- | --- | --- |
| chinese 1 | 0.992 | 4.72 | 2003 | 4.72 |
| chinese 2 | 0.997 | 1.82 | 2003 | 1.82 |
| chinese 3 | 0.995 | 3.63 | 2003 | 3.63 |
| chinese 4 | 0.998 | 1.36 | 2003 | 1.36 |
| chinese 5 | 0.999 | 0.64 | 2003 | 0.64 |
| wos 1 | 0.444 | 50.00 | 2006 | 41.90 |
| wos 2 | 0.864 | 26.19 | 2014 | 26.19 |
| wos 3 | 0.840 | 26.19 | 2014 | 26.19 |
| wos 4 | 0.913 | 25.00 | 2006 | 17.78 |

Rho is Spearman correlation between the two annual prevalence series on years where both denominators are positive. pp means percentage points; n≥10 uses the all-record annual count. Chinese peak-year sets were unchanged. For WoSCC community 2 (evidence synthesis, trials, and post-stroke depression), the peak changed from 2025 to 2016. Restricting to years with at least ten records, community 1 changed from a 2012 peak to a tie in 2009/2012; community 3 changed from 2019 to 2014; community 4 changed from 2011 to a tie in 2011/2013/2017. The largest WoSCC difference was 50 percentage points in a four-record year, and remained 41.90 points after the n≥10 restriction. These are material differences in time-profile interpretation, not evidence of a change in the underlying clinical field. Figure 9 retains the all-record descriptive denominator with an explicit caveat; the conditional curves in Figure S1 expose the alternative rather than replacing the primary view without explanation.

Figure S1. Annual community-term prevalence under all-record and available-keyword denominators. Blue solid lines use all records; orange dashed lines condition on keyword availability. Communities use the numbering of the primary manuscript. Gaps indicate unavailable denominators. Both lines retain the same observed numerators and fixed community membership; differences therefore quantify denominator dependence, not clinical effects.

## S9. Computational verification

The custom workflow was selected to preserve record-level source identifiers, exact deduplication and mapping logs, complete graph exports, explicit thresholds, deterministic layouts, and sensitivity outputs across separate Chinese and WoSCC corpora. Input and configuration file identities are retained in the accompanying technical record (Technical_File_Identities.tsv).

An exact pinned Python environment passed 58 tests. R/igraph independently checked annual totals, node/edge counts, frequency, degree, strength, density, components, weighted betweenness, community modularity, seed membership and adjusted Rand indices, and Leiden sensitivity: 107 checks passed with zero failures. This demonstrates computational consistency for checked quantities; it does not remove database, selection, normalization, or interpretive bias.

Available CiteSpace workspaces used different corpora or time windows and were not used as a benchmark. The original executable and command were not completely retained. A later validation records its own environment and command and does not retrospectively establish the original environment. The current record-level keyword reconstruction reproduces every primary eligible node frequency and edge weight before the alternative terminology mapping is applied; the current runtime is Python 3.12.13, NumPy 2.4.6, pandas 3.0.5, and NetworkX 3.6.1. Primary community modularity and silhouettes were also reproduced. The analysis scripts distinguish primary outputs, verification runs, and alternative mappings.

## S10. Citation-network scope

The WoSCC corpus contained usable cited-reference data for 720 of 721 records and 36,153 cited references. The Chinese exports and final Chinese analytical records did not contain record-level reference lists. Therefore, comparable Chinese and WoSCC co-citation or bibliographic-coupling networks could not be constructed. A WoSCC-only network would answer a narrower, single-corpus question and was not added to this cross-database design.
